# Activity-based protein profiling reveals dynamic substrate-specific cellulase secretion by saprotrophic basidiomycetes

**DOI:** 10.1186/s13068-022-02107-z

**Published:** 2022-01-17

**Authors:** Nicholas G. S. McGregor, Casper de Boer, Mikhaaeel Santos, Mireille Haon, David Navarro, Sybrin Schroder, Jean-Guy Berrin, Herman S. Overkleeft, Gideon J. Davies

**Affiliations:** 1 York Structural Biology Laboratory, Department of Chemistry, The University of York, Heslington, YO10 5DD York UK; 2 Leiden Institute of Chemistry, Leiden University, Einsteinweg 55, 2300 RA Leiden, The Netherlands; 3 UMR1163 Biodiversité et Biotechnologie Fongiques, Faculté des Sciences de Luminy, INRAE, Aix Marseille Univ, 13288 Marseille, France; 4 Polytech Marseille, Aix Marseille Univ, 13288 Marseille, France

**Keywords:** Cellulase, Glycoside hydrolase, Activity-based protein profiling, Cyclophellitol, Basidiomycete, Biomass, Secretome, Fluorescence, Enzyme identification, *Pichia pastoris*, Activity-based probe, Filamentous fungi, Enzyme secretion, Kinetics

## Abstract

**Background:**

Fungal saccharification of lignocellulosic biomass occurs concurrently with the secretion of a diverse collection of proteins, together functioning as a catalytic system to liberate soluble sugars from insoluble composite biomaterials. How different fungi respond to different substrates is of fundamental interest to the developing biomass saccharification industry. Among the cornerstones of fungal enzyme systems are the highly expressed cellulases (*endo*-β-glucanases and cellobiohydrolases). Recently, a cyclophellitol-derived activity-based probe (ABP-Cel) was shown to be a highly sensitive tool for the detection and identification of cellulases.

**Results:**

Here we show that ABP-Cel enables *endo*-β-glucanase profiling in diverse fungal secretomes. In combination with established ABPs for β-xylanases and β-d-glucosidases, we collected multiplexed in-gel fluorescence activity-based protein profiles of 240 secretomes collected over ten days from biological replicates of ten different basidiomycete fungi grown on maltose, wheat straw, or aspen pulp. Our results reveal the remarkable dynamics and unique enzyme fingerprints associated with each species substrate combination. Chemical proteomic analysis identifies significant arsenals of cellulases secreted by each fungal species during growth on lignocellulosic biomass. Recombinant production and characterization of a collection of probe-reactive enzymes from GH5, GH10, and GH12 confirm that ABP-Cel shows broad selectivity towards enzymes with *endo*-β-glucanase activity.

**Conclusion:**

Using small-volume samples with minimal sample preparation, the results presented here demonstrate the ready accessibility of sensitive direct evidence for fungal enzyme secretion during early stages of growth on complex lignocellulosic substrates.

**Supplementary Information:**

The online version contains supplementary material available at 10.1186/s13068-022-02107-z.

## Introduction

The diversity of biomass sources, containing different compositions of various polysaccharides, such as hemicelluloses [1] and pectins [2], presents a challenge to saprotrophs. The organism must possess the right combination of enzyme systems and molecular logic to efficiently sense and degrade the various linkages holding the material together. Identifying the right saprotrophic organism(s) to degrade industrially available biomass presents a match-making challenge in bioprocess development. It is clear that no single biomass-degrading organism is proficient at digesting all types of biomass, and that a variety of species will be needed to facilitate the utilization of the various agricultural biomass streams that are available today [3, 4]. Tools to rapidly screen different fungi for their ability to recognize and grow on distinct complex carbohydrate-based substrates, particularly broadly accessible tools amenable to efficient small-scale enzyme detection and identification, are needed to enhance enzyme discovery and species characterization.

Lignocellulosic biomass is a highly variable complex composite material assembled from non-carbohydrate and carbohydrate polymers, including cellulose, hemicelluloses (primarily β-xylans, β-mannans, and non-cellulosic β-glucans), pectins, and lignin [1, 5–7]. The carbohydrate components of this biomass represent the bulk of the chemical potential energy available to saprotrophic organisms. Thus, saprotrophs produce large arsenals of carbohydrate-degrading enzymes when growing on such substrates [8–10]. These arsenals typically include polysaccharide lyases, carbohydrate esterases, lytic polysaccharide monooxygenases (LPMOs), and glycoside hydrolases (GHs) [11]. Of these, GHs and LPMOs form the enzymatic vanguard, responsible for generating soluble fragments that can be efficiently absorbed and broken down further [12].

The identification, usually via bioinformatic analysis of comparative transcriptomic or proteomic data, of carbohydrate-active enzymes (CAZymes) that are expressed in response to specific biomass substrates is an essential step in dissecting biomass-degrading systems. Due to the underlying molecular logic of these fungal systems, detection of carbohydrate-degrading enzymes is a useful indicator that biomass-degrading machinery has been engaged [9]. Such expression behaviour can be hard to anticipate and methods of interrogation generally have low throughput and long turn-around times. Indeed, laborious scrutiny of model fungi has consistently shown complex differential responses to varied substrates [13–15]. Much of this complexity still remains obscure, presenting a hurdle in saccharification process development [16]. In particular, while many ascomycetes, particularly those that can be cultured readily at variable scales, have been investigated in detail [17, 18], only a handful of model organisms from the diverse basidiomycetes have been studied, with a focus on oxidase enzymes [19, 20].

Made possible by the recent sequencing of various basidiomycete genomes [21, 22], activity-based protein profiling (ABPP) offers a rapid, small-scale method for the detection and identification of specific enzymes within the context of fungal secretomes [23, 24]. ABPP revolves around the use activity-based probes (ABPs) to detect and identify specific probe-reactive enzymes within a mixture [25]. ABPs are covalent small-molecule inhibitors that contain a well-placed reactive warhead functional group, a recognition motif, and a detection handle [26]. Cyclophellitol-derived ABPs for glycoside hydrolases (GHs) use a cyclitol ring recognition motif configured to match the stereochemistry of an enzyme’s cognate glycone [27, 28]. They can be equipped with epoxide [29], aziridine [30], or cyclic sulphate [31, 32] electrophilic warheads, which all undergo acid-catalysed ring-opening addition within the active site [33]. Detection tags have been successfully appended to the cyclitol ring [29] or to the (*N*-alkyl)aziridine, [34] giving highly specific ABPs. The recent glycosylation of cyclophellitol derivatives has extended such ABPs to targeting retaining *endo*-glycanases, opening new chemical space. ABPs for *endo*-α-amylases, *endo*-β-xylanases, and cellulases (encompassing both *endo*-β-glucanases and cellobiohydrolases) have been developed [35–37]. Initial results with these probes have demonstrated that their sensitivity and selectivity is sufficient for glycoside hydrolase profiling within complex samples.

**Fig. 1:**
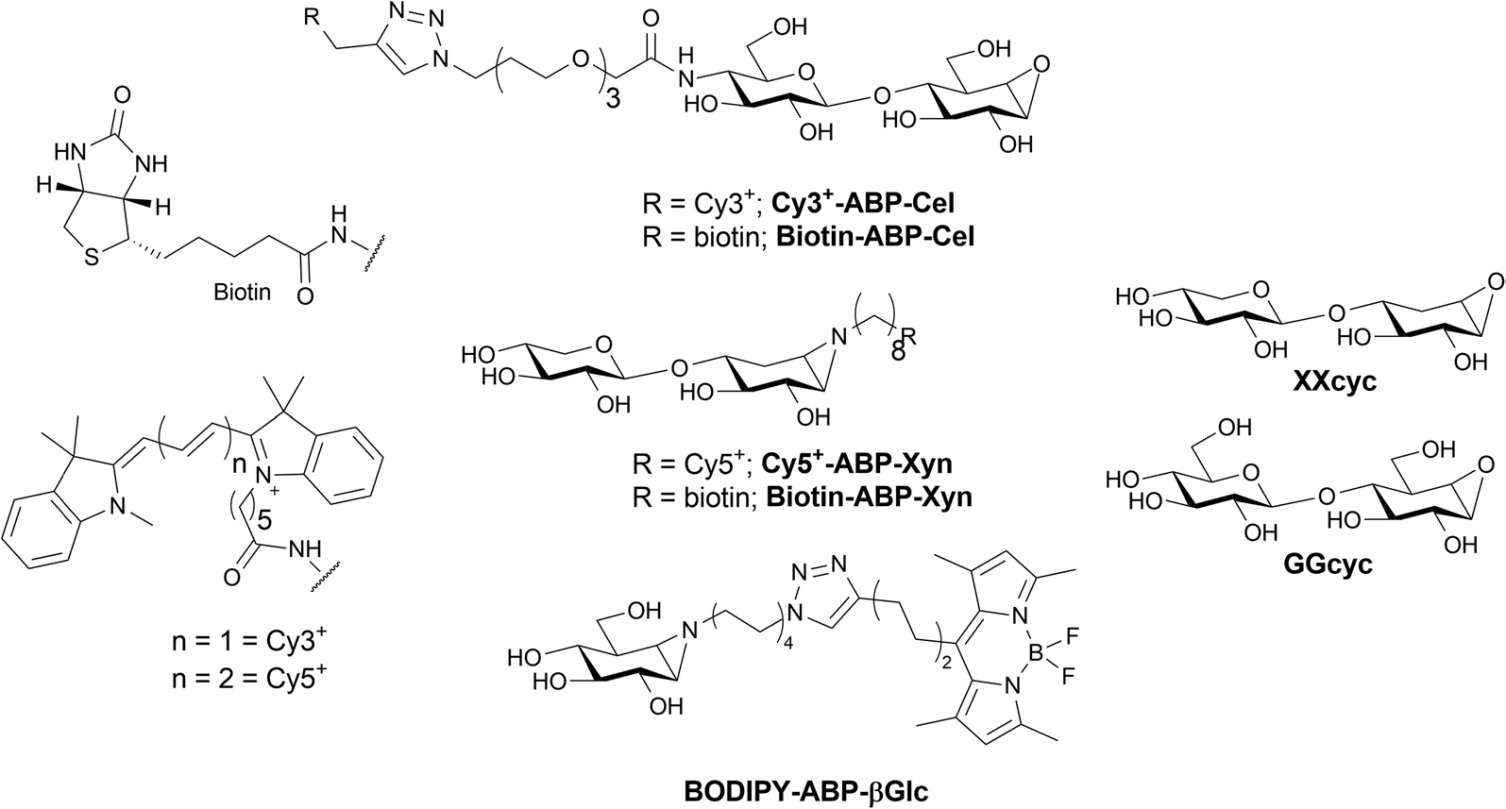
Structures and given names (bold) of probes and inhibitors used in this study

To profile fungal enzymatic signatures, we sought to combine multiple probes that target broadly distributed biomass-degrading enzymes (Fig. 1). Cellulases and β-glucosidases are known to be some of the most broadly distributed and most highly expressed components of enzymatic plant biomass-degrading systems [11, 38]. Among the hemicellulose-degrading enzymes, GH10 xylanases are broadly distributed, being found in every kingdom of life [5, 39]. Using validated probes targeting cellulases, xylanases, and β-glucosidases, we report here the results from a rapid, small-scale multiplex in-gel fluorescence-based ABPP assay. We demonstrate the ability of this assay to detect and identify diverse enzymes that are secreted by a collection of 10 different basidiomycete fungi over time under different growth conditions. Recombinant production of a collection of detected GH family representatives shows correlation between probe reactivity and enzyme activity.

## Results and discussion

### Preparation of basidiomycete secretomes

Ten fungi were selected from the “Centre International des Ressources Microbiennes” (CIRM) collection for profiling on the basis that are all known basidiomycete saprotrophs with sequenced genomes (Additional file 11: Table S1). These included *Abortiporus biennis* [40], *Fomes fomentarius* [41], *Hexagonia nitida*, *Leiotrametes menziesii* [42], *Polyporus brumalis* [43], *Trametes ljubarskyi* [44], *Trametes gibbosa* [45], *Pycnoporus sanguineus* [46], *Leiotrametes* sp. 1048 [47], and *Trametes meyenii* [47]. Annotated genomes for each of these are available publicly through JGI Mycocosm [22].

Each fungus was cultured in a general minimal medium (see “Experimental”) supplemented with either wheat straw (an abundant monocot lignocellulosic substrate rich in arabinoxylan) [48], aspen pulp (a woody dicot biomass rich in glucuronoxylans and mannans) [49, 50], or maltose (a control substrate which does not induce biomass-degrading enzyme production [21]). The use of wheat straw and aspen pulp facilitates comparison to previous integrative omics studies of basidiomycetes [46, 51]. Duplicate time-course cultures were grown from individual mycelial starter cultures for 10 days to give ample time for substrate recognition and digestion. The use of small, baffled flasks shaking at 120 rpm minimized, but likely did not eliminate, mechanical cell lysis while promoting aeration. Secretomes collected at days 3, 5, 7, and 10 from maltose and aspen-grown cultures developed minimal colour over time, varying from clear to light yellow. Wheat straw cultures developed strong yellow-to-brown colour over the course of culturing, generally giving a denser, more aggregated mycelium.

### Fluorescence-based secretome profiling

**Fig. 2:**
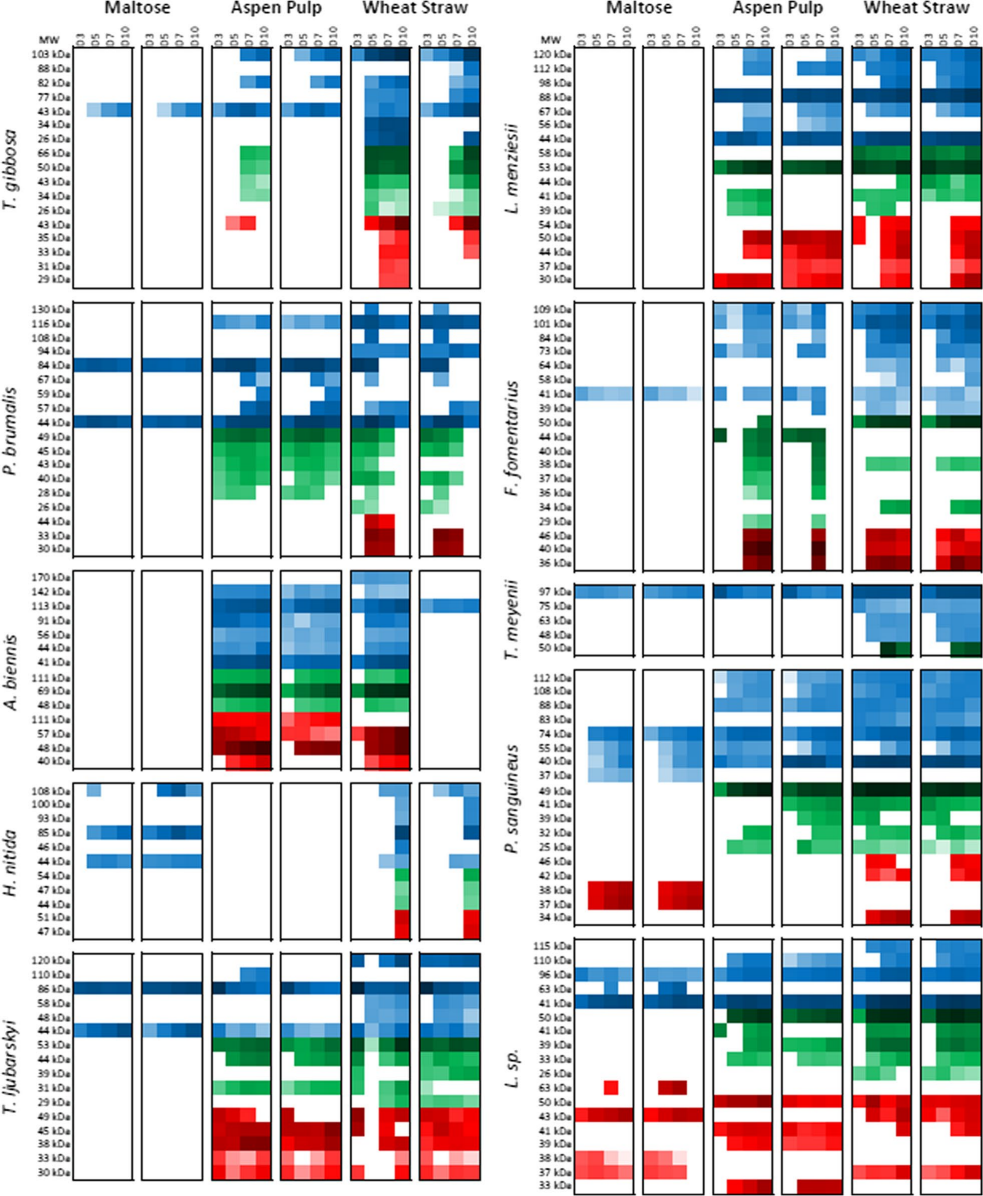
Quantified ABP fluorescence of bands detected following SDS-PAGE of basidiomycete secretomes stained with BODIPY-ABP-βGlc (blue), Cy3^+^-ABP-Cel (green), and Cy5^+^-ABP-Xyn (red). The intensity of the colour of each square represents the integrated fluorescence for the observed bands on a log scale from white (< 100,000 counts) to full colour (at ~ 4,000,000 counts) to black (> 250,000,000 counts). The apparent molecular weight of the observed band is given to the left of each row of squares. Data are organized by species (abbreviated to the left of each collection of squares) and by substrate (top). Two sets of four time points (D3, D5, D7, and D10, noted above each column of squares) represent two biological replicates measured for each substrate species combination

The inclusion of maltose in the complex substrate cultures allows rapid early expansion of biomass, typically being consumed over the course of the first two days of culture [21]. Thus, it was expected that day 3 secretomes would be dominated by early oxidative enzymes as observed previously [8, 52] and that cellulose- and hemicellulose-degrading enzymes would be detected at later time points, with increasing signal over time. Incubation of each of our 240 secretome samples (centrifuged and filtered) with the triplex probe mixture for 1 h followed by SDS-PAGE separation and fluorescence imaging yielded a collection of visual species-specific enzyme profiles (Additional file 11: Figs. S1–S10). Qualitative inspection of these images reveals clear signatures of biomass recognition in most cases, with differential glycoside hydrolase expression between each substrate and significant variation over time. Surprisingly, the gel images clearly show the presence of low levels of cellulase secretion following only three days of culturing in many cases, particularly *A. biennis*, *P. brumalis*, and *L. menziesii*. Background interference can be observed in the Cy5^+^ channel in many of the wheat straw secretomes. This interference correlates with the darkness of secretome colour, visible as a tan-coloured streak in the gel following separation of some of the most darkly coloured, notably *P. brumalis*, wheat straw-grown secretomes. We were not able to remove this material via selective precipitation or adsorption (e.g. using PVPP) without losing proteins of interest, so xylanase detection was partially obscured in some cases. To quantify relative enzyme levels and provide good estimates of enzyme molecular weight, fluorescent lane profiles were determined for each channel and peaks were integrated with subtraction of a rolling ball baseline. Integrated peak intensities were then plotted over time on a log scale to show enzyme concentration variation for each detected band across ~ 3.5 orders of magnitude (Fig. 2).

Each species showed a distinct pattern of behaviour. *T. gibbosa* took 5–7 days to initiate enzyme secretion. Following this extended lag phase, it showed a strong response to wheat straw, producing an array of apparent cellulases, glucosidases, and xylanases. Its response to aspen was much more muted, with exceptionally weak cellulase expression in one replicate and weak glucosidase expression in both. *P. brumalis* recognized both substrates rapidly, showing significant cellulase expression at 3 days. Interestingly, cellulase and glucosidase levels peaked at days 5–7 in all *P. brumalis* cultures, with xylanases only detected in the wheat straw culture. Strikingly, the *P. brumalis* secretome decayed rapidly following its day 5–7 peak. A*. biennis* showed clear strong recognition of both substrates after 3–5 days, secreting xylanases, cellulases, and glucosidases. A major xylanase band at ~ 57 kDa was lost over time in the aspen culture but increased over time in the wheat straw culture. An apparent xylanase band at 111 kDa may be a β-xylosidase, given the high molecular weight of GH3 xylosidases and the known tendency of this probe to cross-react [35]. *H. nitida* did not appear to strongly recognize any of the substrates, though a mixture of enzymatic signatures could be detected in the wheat straw cultures at the 10 day mark, suggesting that longer culturing is needed for the full development of *H. nitida* under these conditions. *T. ljubarskyi* showed remarkably complex behaviour. When grown on aspen pulp, it rapidly produced an array of xylanases, some of which grew over time while others decayed. Cellulase levels were low, but consistently rose. When grown on wheat straw, it rapidly produced a high level of cellulases and xylanases. This was then followed by a rapid loss of most of these enzymes, correlated with a notable increase in background fluorescence in the Cy5^+^ channel. Slow background decay and restoration of most of these hydrolases followed with the two replicates showing different enzyme levels. We speculate that this is indicative of variable growth behaviour, oscillating between oxidative and hydrolytic catabolism. *L. menziesii* showed rapid wheat straw recognition and slower aspen recognition, characterized by low levels of xylanase, and high levels of cellulase and glucosidase production. Interestingly, the higher molecular weight cellulase band was only observed during growth on wheat straw. *F. fomentarius* recognized substrate rapidly, producing detectable cellulase and glucosidase at day 3. Like *T. ljubarskyi*, it showed the remarkable ability to temporarily eliminate its diverse complement of secreted glycoside hydrolases, particularly evident in the aspen cultures at day 5 in the first replicate and day 10 in the second replicate. The wheat straw cultures showed more consistent behaviour, with a steady increase in xylanase, cellulase, and glucosidase levels over time. *T. meyenii* did not appear to recognize the aspen pulp, but did recognize the wheat straw after 7 days, expressing a high level of a singular cellulase and a small host of apparent glucosidases. *P. sanguineus* produced the most diverse complement of enzymes, producing high levels of cellulase, particularly after 5 days. Diverse glucosidases and xylanases were also detected, particularly in the wheat straw secretome. *P. sanguineus* was the only organism that produced an apparent xylanase in the maltose culture, though this was a different molecular weight from those detected during growth on biomass. Similarly, *Leiotrametes* sp. 1048 produced consistently high levels of cellulase and a diverse collection of xylanases and glucosidases following 5 days of growth on either wheat straw or aspen pulp substrates. Together, these results show the diversity of fungal strategies for biomass degradation and highlights the challenge of identifying apparently productive fungus–substrate interactions. Taking rising cellulase and xylanase titres as an indicator of a productive interactions between fungus and substrate, we can observe clear preferences of *T. gibbosa*, *L. menziesii*, *Leiotrametes* sp. 1048, and *P. sanguineus* for wheat straw, while *T. ljubarskyi* and *A. biennis* showed an apparent preference for aspen pulp.

### Chemical proteomic identification of putative cellulases

**Fig. 3:**
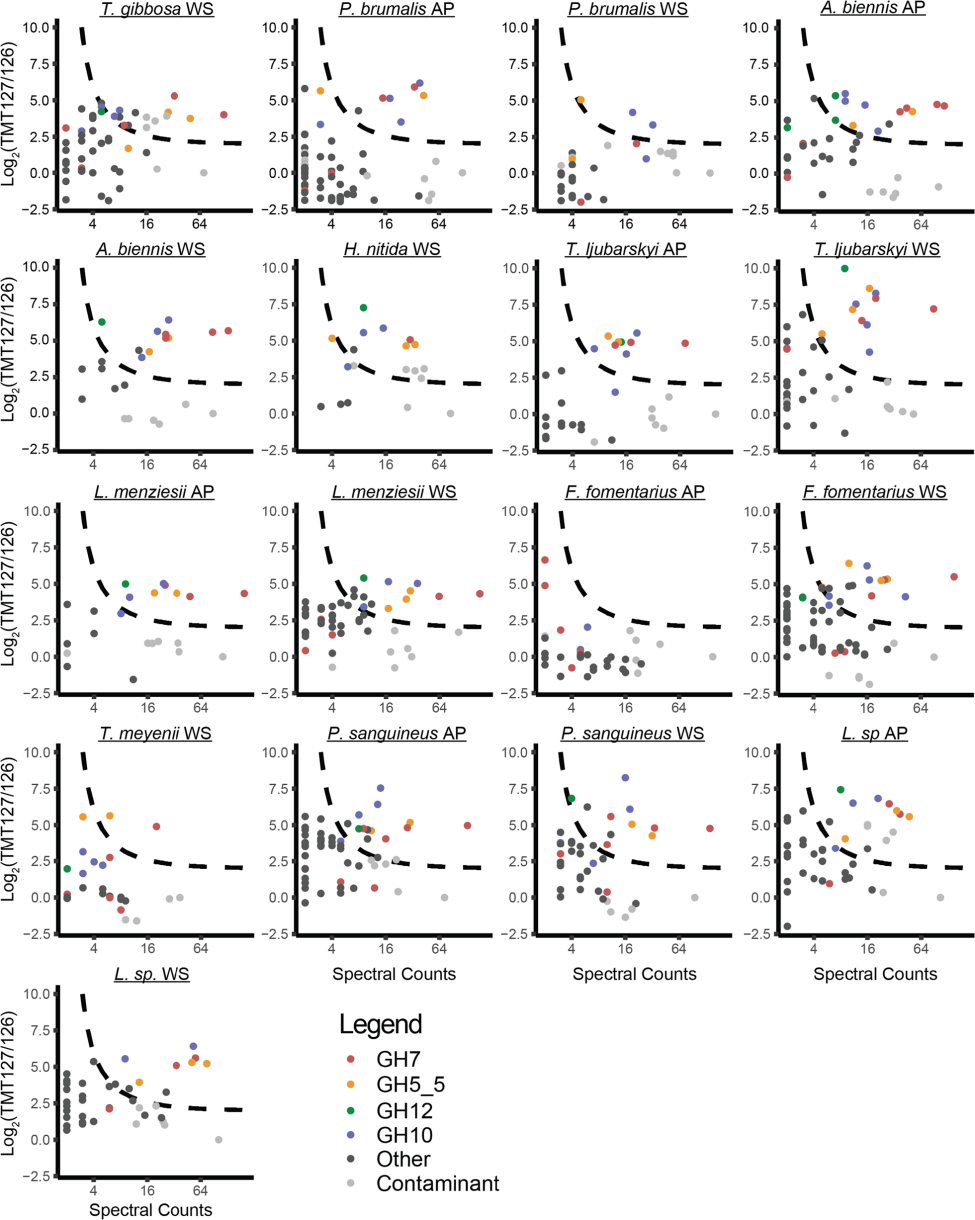
CAZymes identified in the pulldown from the day 10 secretomes using biotinylated ABP-Cel. Each plot shows a point for each protein detected (minimum 2 peptides at 1% FDR) in the day 10 secretome listed above the plot (AP = aspen pulp, WS = wheat straw). The *x*-axis is the number of spectra collected for peptides assigned to each protein (log_2_ scale) and the *y*-axis is the log_2_TMT127/126 ratio (127 = labelled, 126 = vehicle control) calculated by Scaffold for the protein, normalized using the TMT ratio of streptavidin. Points corresponding to putative retaining *endo*-β-glucanases/xylanases are coloured according to glycoside hydrolase family; other proteins are coloured dark grey. Detected contaminants not derived from the fungi under study (e.g. streptavidin, trypsin, keratins) are coloured light grey. A hyperbolic hit cut-off line is shown as a black dashed line with lower limits at 2 spectral counts and a 127/126 ratio of 4. Points found above this line are both well detected in the pulldown sample and depleted in the vehicle control. Source data (Excel format) can be found in Additional files 1, 2, 3, 4, 5, 6, 7, 8, 9, 10 and 11. Plots were prepared using ggplot2

Interested in the identities of the apparent cellulases in the basidiomycete secretomes and the identification of novel *endo*-β-glucanases, we used the biotinylated derivative of ABP-Cel (Biotin-ABP-Cel) to label the cellulases found in the day 10 secretomes. Labelled enzymes (and a negative control treated with vehicle) were pulled down from 2 mL of secretome using streptavidin beads and peptides were generated via on-bead digestion using trypsin. To assist in the filtration of background signals, while facilitating the throughput needed to analyse 17 samples using the relatively small sample volume available, we labelled negative control samples with TMT^2^-126 and probe-treated samples with TMT^2^-127. These were mixed 1:1 prior to separation and analysis. Thus, orthogonal signals of spectral counts (indicative of overall abundance in the pulldown) and TMT ratios (indicative of selective enrichment in the pulldown) were collected for each identified protein in a single 1-h run. This enabled the identification of both major and minor probe-reactive secretome components (Fig. 3, Additional files 1, 2, 3, 4, 5, 6, 7, 8, 9 and 10). Contaminating proteins common to both probe-treated and negative control samples (i.e. trypsin, streptavidin) were generally found to have TMT ratios close to 1, indicating that a TMT 127/126 ratio close to 1 is a robust basis on which to exclude background signals (Fig. 3).

In all cases, the strongest hits from ABP-Cel were putative cellulases or xylanases from families GH7, GH5_5, GH10, and GH12. The detected enzymes represent a majority of the total predicted GH5_5 (85% of the annotated genes across all 10 fungi) and GH7 (83% of annotated genes) cellulases annotated in the genomes of each fungus (Table 1), indicating that this method is suitable for the broadly specific detection of core cellulases. Similarly, our method achieved reasonably comprehensive detection of annotated GH10 enzymes, identifying 66% of the annotated genes. GH12 enzymes, however, gave a significantly lower detection rate (35% of annotated genes). All of the GH7 enzymes detected are close homologues of known, and well-characterized, cellobiohydrolases [53, 54]. Similarly, the GH5_5 enzymes that were detected are homologues of well-known *endo*-β(1,4)-glucanases that show specificity towards linear glucans, such as carboxymethylcellulose (CMC, an artificial soluble cellulose derivative) or mixed-linkage β-glucan (bMLG) [55, 56]. GH10 enzymes are only known to be *endo*-β(1,4)-xylanases, though weak *endo*-β(1,4)-glucanases activity has been reported in the family [57]. GH12 enzymes have been reported to have variable specificities, recognizing linear or branched (i.e. xyloglucan) β(1,4)-glucans [58, 59]. This divergent substrate specificity within GH12 may explain the low number of detected GH12 enzymes, though low levels of GH12 expression during growth on wheat straw and aspen pulp, reduced detection efficiency due to their low molecular weight, or generally poor reactivity of the probe with GH12 enzymes may also contribute.

**Table 1:**
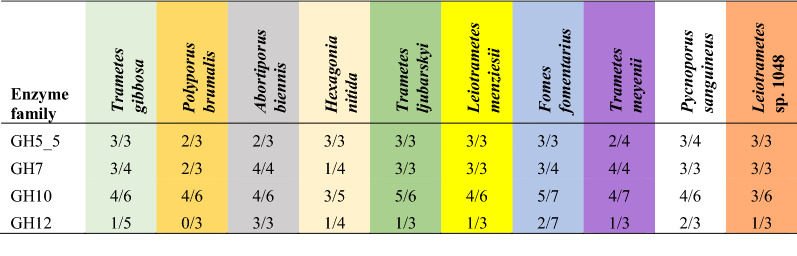
Detected hits from pulldown experiments compared to the total number of GH family members in each fungal genome

Each cell contains (the number of detected GH family members)/(the number of annotated GH family members in the genome)

Several unexpected proteins also gave significant hits. The most abundant and consistently detected of these were members of GH5_7 [11], a well-characterized subfamily of *endo*-β-mannanases. Other less frequent marginal detections included a handful of enzymes from GH families 6 (inverting), 28 (inverting), 74 (inverting), and 152 (thaumatin like), as well as a glutamic protease (eqolisin like). These detection events may point to a weak broader non-specific reactivity with enzymes containing activated glutamate residues. However, such non-specific reactivity is not in line with general epoxide reactivity, which favours cysteine residues [60]. Larger datasets are needed to explore the significance and consistency of the marginal detections observed in pulldown experiments using ABP-Cel.

Comparing the predicted molecular weights (MWs) of proteomic hits with observed bands on SDS-PAGE presents a challenge due to the known tendency of fungi to glycosylate or proteolyse secreted protein and the complexity of the band patterns on each gel. However, we attempted some inference considering both expected correlations between band intensity and spectral count (SC), and between theoretical and apparent MWs. Considering the case of the *P. sanguineus* wheat straw secretome, we observed minor bands at 25, 32 and 41 kDa and a strong broad band at 49 kDa. The only hit close to 25 kDa is a GH12 weak hit (4 SCs) with a predicted MW of 26 kDa. No hit could be readily matched to the observed 32 kDa band, perhaps indicating that it was either undetected or a result of proteolysis. The dominant 49 kDa band matches the theoretical MW of a GH7 cellobiohydrolase, which gave the single strongest signal observed in the proteomic data (142 SCs). However, considering the remainder of the observed hits, most of these are not apparently resolved on SDS-PAGE. We conclude from this that analysis of in-gel fluorescence bands is generally not sufficient to assess the diversity of the often microheterogeneous *endo*-β(1,4)-glucanase components of basidiomycete secretomes, necessitating routine chemical proteomic analysis for the assessment of molecular diversity. Alternative separation techniques (e.g. liquid chromatography, capillary electrophoresis) may offer the resolution needed to better distinguish enzymes with such similar apparent molecular weights.

### Testing enzyme specificity via recombinant production

To assess the specificity of ABP-Cel for cellulases, we sought to determine the true substrate specificities of representatives of the detected enzyme clades. Towards this end, pure enzyme samples were needed. Thus, we selected a GH5_5 enzyme (LsGH5_5A; 27 spectral counts (SCs), TMT ratio (127/126) = 52), a GH10 enzyme (LsGH10A; 20 SCs, 127/126 = 93), a GH12 enzyme (TlGH12A; 10 SCs, 127/126 = 31), and a GH5_7 enzyme (LsGH5_7A; 3 SCs, 127/126 = 52) for recombinant production. Homologues of all of these were detected as components above the cut-off in pulldowns from multiple fungal species. Each sequence was codon optimized for *P. pastoris*, synthesized and cloned into pPICZα with a C-terminal 6×His tag, and native signal peptide replaced with the α-factor secretion tag. They were transformed into *Pichia pastoris* X-33 and produced under methanol induction in shake flasks, giving high yields of electrophoretically pure enzymes (Additional file 11: Fig. S11).

To establish a basis for an inhibition assay we measured hydrolytic activity towards 4-methylumbelliferyl cellobioside (4MU-GG). LsGH5_5A, LsGH10A, and TlGH12A all showed detectable hydrolytic activity towards 4MU-GG (Additional file 11: Table S2, Fig. S12), while LsGH5_7A did not. As an initial test of specificity, we compared activity towards 4MU-GG and 4-methylumbelliferyl xylobioside (4MU-Xyl2), finding no detectable activity towards 4MU-Xyl2 among LsGH5_5A and TlGH12A, and a strong preferential activity towards 4MU-Xyl2 for LsGH10A (Additional file 11: Table S2). Using 4MU-GG as substrate, we measured inhibition of LsGH5_5A, LsGH10A, and TlGH12A over time by glucosyl-β(1,4)-cyclophellitol [36] (GGcyc) at inhibitor concentrations as high as 50 μM under optimal buffer conditions (see Additional file 11: Figs. S13 and S14 for effects of buffer and pH on enzyme activity). This revealed clear time-dependent inhibition of LsGH5_5A, TlGH12A, and LsGH10A by GGcyc (Additional file 11: Figs. S15–S17) with similar performance constants (*k*_i_/*K*_I_, Additional file 11: Table S3), providing an explanation for the comparable detections of GH5, GH10, and GH12 enzymes in the pulldown. Comparison to inhibition with xylosyl-β(1,4)-xylocyclophellitol [35] (XXcyc) provided further evidence, the LsGH5_5A and TlGH12A are specific *endo*-β-glucanases, while LsGH10A is a specific *endo*-β-xylanase (Additional file 11: Table S3). The move from GGcyc to ABP-Cel somewhat reduced potency towards TlGH12A compared to GGcyc and had no apparent impact on reactivity with LsGH5_5A. In contrast, Biotin-ABP-Xyn bound to LsGH10A non-covalently with 21 nM affinity, but no covalent inhibition was discernable after 1 h, similar to previously reported behaviour among GH10 xylanases [35]. Thus, the addition of Biotin-ABP-Xyn to a secretome-labelling reaction can serve as a way to “block” GH10 active sites, but does not efficiently label xylanases on the time scales used in this assay, preventing pulldown and identification of xylanases using Biotin-ABP-Xyn.

To assess enzyme polysaccharide specificity, reducing end-based activity assays were performed with a panel of β-glucan, β-xylan, and β-mannan substrates (Table 2). TlGH12A showed strong activity towards CMC and bMLG with only weak xyloglucanase activity, suggesting that this is indeed a cellulase-type GH12. LsGH10A showed strong activity towards wheat arabinoxylan (wAX), with weak activity towards bMLG and CMC, confirming that it does have cellulase activity, though it is primarily a xylanase. LsGH5_7A showed dominant activity towards carob galactomannan (cGM), in line with previous observation that GH5_7 enzymes are β(1,4)-mannanases [61]. LsGH5_7A also displayed weak activity against CMC and bMLG, a previously unreported phenomenon possibly rationalizing the observed weak hit in the pulldown. Finally, LsGH5_5A showed dominant activity towards CMC and bMLG with no detectable xyloglucanase activity, confirming that it is a cellulase. Thus, we conclude that ABP-Cel is selective towards enzymes that recognize glucans, allowing the identification of a list of probable cellulases. However, detectable reactivity with ABP-Cel should not be taken as sufficient evidence to assign enzyme specificity, as detected enzymes may be either *endo*-glucanases or *endo*-xylanases.

**Table 2:** Enzyme specificity

Enzyme	bMLG	CMC	tXyG	wAX	cGM
LsGH5_5A	19 ± 2	11 ± 1	< 0.01	< 0.01	< 0.01
LsGH5_7A	0.06 ± 0.01	0.04 ± 0.01	< 0.01	< 0.01	14 ± 2
LsGH10A	< 0.01	0.05 ± 0.01	< 0.01	8 ± 1	< 0.01
TlGH12A	20 ± 2	13 ± 1	0.04 ± 0.01	< 0.01	< 0.01

Specific activity values (μmol/min/mg) measured for LsGH5A, LsGH5B, LsGH10A, and TlGH12A acting on 1 mg/mL barley mixed-linkage glucan (bMLG), carboxymethylcellulose (CMC), tamarind xyloglucan (tXyG), wheat arabinoxylan (wAX), or carob galactomannan (cGM)

## Conclusions

Here we have presented an ABPP-based method for the rapid detection of multiple cellulose- and xylan-degrading glycoside hydrolases in fungal secretomes. This method enables time-resolved studies of fungal enzyme secretion in response to lignocellulosic substrates using small-volume samples. Applying this method to basidiomycete secretomes, we have shown that most of the fungi in this study produce significant complements of cellulases, glucosidases, and xylanases in response to different sources of lignocellulosic biomass. Furthermore, we have shown that the secreted enzyme complements can vary significantly over time, being completely degraded and restored on the timescale of days. Using chemical proteomic methods, we have identified a collection of putative cellulases and shown, through recombinant production and characterization, that they do, in fact, possess *endo*-glucanase activity. Despite this, we find that the major detected enzymes may either be *endo*-glucanases or *endo*-xylanases. Thus, the function of enzymes identified using ABP-Cel should be assigned with consideration of the functions of characterized homologues or supplemental functional assays of purified enzymes. We expect that the development of improved ABPs for other *endo*-glycanases built on the ABP-Cel architecture will enable ABPP-based specificity determination.

## Experimental

All chemicals were purchased from Sigma unless otherwise specified.

### Design and synthesis of cyclophellitol-derived probes

For multiplex fluorescent ABPP, three probes, each bearing a different fluorophore and a different combination of recognition motif and reactive warhead, were used. JJB376, an established *N*-alkyl aziridine probe bearing a BODIPY-FL [62] tag was used to label β-glucosidases [34]. ABP-Xyn, an established *N*-alkyl aziridine probe bearing a Cy5^+^ tag, was used to label *endo*-β-xylanases [35]. *Endo*-β-glucanase probe CB644 was prepared through click modification of ABP-Cel with Cy3^+^ alkyne in place of previously reported Cy5^+^ alkyne [36].

### Basidiomycete culture preparation and secretome collection

The strains *Abortiporus biennis* BRFM 1215 (*A. biennis*), *Fomes fomentarius* BRFM 1323 (*F. fomentarius*), *Hexagonia nitida* BRFM 1328 (*H. nitida*), *Leiotrametes menziesii* BRFM 1557 (*L. menziesii*), *Polyporus brumalis* BRFM 985 (*P. brumalis*), *Trametes ljubarskyi* BRFM 957 (*T. ljubarskyi*), *Trametes gibbosa* BRFM 952 (*T. gibbosa*), *Pycnoporus sanguineus* BRFM 902 (*P. sanguineus*), *Leiotrametes* sp. BRFM 1048 (*L.* sp.), and *Trametes meyenii* BRFM 1361 (*T. meyenii*) were obtained from the CIRM-CF collection (International Centre of Microbial Resources dedicated to Filamentous Fungi, INRA, Marseille, France). All strains were identified by morphological and molecular analysis of Internal Transcribed Spacer (ITS) sequences. The strains were maintained on malt agar slants at 4 °C.

Five discs (5 mm each) of fungal mycelium grown on malt agar plates were used to inoculate Roux flasks containing 100 mL of medium (glucose 10 g/L; bactopeptone 20 g/L; yeast extract 1 g/L). After incubation during 15 days at 30 °C without shaking, the fungal mycelium was ground (ultraturax 10,000 rpm, 60 s) in 50 mL of purified water (MilliQ, Millipore). Five mL of this suspension was used for the inoculation of each 250-mL baffled Erlenmeyer flasks containing 100 mL medium with 2.5 g/L of maltose as a starter (except for the maltose control condition; 20 g/L), 1.842 g/L of diammonium tartrate as a nitrogen source, 0.5 g/L yeast extract, 0.2 g/L KH_2_PO_4_, 0.0132 g/L CaCl_2_/2H_2_O, and 0.5 g/L MgSO_4_/7H_2_O, and as a main carbon source, 15 g/L (dry weight) of ball-milled wheat straw (*Triticum aestivum*) or Wiley-milled aspen (*Populus grandidentata*). Cultures were incubated in the dark at 30 °C with shaking at 120 rpm. 5 mL of each culture was sampled at 3, 5, 7, and 10 days after inoculation and the culture broths (secretomes) were centrifuged, filtered using 0.2-μm polyethersulfone membrane (Millipore) and then stored at − 20 °C until used.

### In-gel fluorescence ABPP assay

Each probe (samples available from Prof. Herman Overkleeft upon request) was dissolved in DMSO at 5 mM and then mixed and diluted with ultrapure water. We prepared a 6× mixture of probes containing 60 μM each of BODIPY-ABP-βGlc, Cy3^+^-ABP-Cel, and Cy5^+^-ABP-Xyn (see Additional file 11: Fig. S18 for probe and inhibitor structures used in this study). Secretome samples were buffered with 0.1 volumes of 1 M NH_4_OAc pH 5.5 to ensure consistent labelling conditions. 25 µL samples of buffered secretome was mixed with 5 μL of 6× probe stock and incubated at 30 °C for 1 h with a heated lid to prevent evaporation. Samples were diluted with 10 µL of 4× SDS-PAGE loading dye, heated to 95 °C for 2 min, and 15 μL of this was separated through 4–15% Criterion gels in an actively cooled Dodeca cell at 200 V for 55 min. Gels were then imaged using the Cy2, Cy3, and Cy5 filter/laser sets in the Typhoon 5 laser scanner. Bands were identified and integrated using ImageQuant (GE Healthcare) with molecular weight estimation based on a Pageruler 10–180 kDa ladder (ThermoFisher), using the bands from 25 to 180 kDa for calibration.

### Pulldown of endo-β-glucanases using ABP-Cel

1.8 mL of buffered day 10 secretomes that showed detectable ABP-Cel signal via fluorescence (17 samples total) was supplemented with 10 μL of 1 mM Biotin-ABP-Cel in DMSO and incubated for 2 h at 30 °C. A separate set of samples treated with 10 μL of DMSO were prepared as negative control. 200 μL of 10× denaturing buffer (40 mM DTT, 2% SDS) was added and the samples were heated to 80 °C for 5 min in a water bath, then cooled to RT. 100 μL of 0.5 M IAA was then added. Following 30 min of incubation at RT in the dark, 9 mL of acetone was added to each sample and they were incubated at − 20 °C overnight. Precipitate (varying in colour from tan to dark orange) was collected by centrifugation at 4000×*g* for 15 min. Supernatant was decanted and the pellets were air dried for ~ 1 h to remove residual acetone. Pellets were dissolved in 40 μL of 10 M urea at RT, transferred to a 0.5 mL lo-bind tube (Eppendorf), then diluted with 360 μL of 0.05% SDS in 50 mM pH 7.4 NaP_i_ buffer. 20 μL of strep mag sepharose suspension was added to each tube and shaken at 25 °C for 1 h. Beads were collected using a magnetic rack and the supernatant was discarded. Beads were washed (resuspended, shaken for 5 min, then collected, and supernatant discarded) with 500 μL of 2% SDS at 40 °C twice, then 500 μL of 2 M urea at rt once, and then with 500 μL of water at rt twice. Beads were finally resuspended in 20 μL of 0.05 M TEAB (Thermo) and supplemented with 0.5 μL of 0.5 μg/μL Trypsin Gold (Promega V5280). Digests were incubated with vigorous shaking overnight at 37 °C. Tubes were then spun down to ensure consistent volume, beads were collected, and the supernatant was supplemented with 2 μL of 20 mg/mL TMT^2^ reagent in absolute ethanol (126 added to negative control and 127 added to probe-treated samples). Labelling reactions were incubated for 1 h at rt; then excess labelling reagent was quenched by addition of 1 μL of 5% hydroxylamine (~ 65 mM final) and incubation for 15 min at rt. 10 μL of TMT^2^-126-labelled negative control and 10 μL of TMT^2^-127-labelled sample peptide solutions were then mixed together and 6 μL was analysed.

### LC–MS analysis of peptides

Peptides from each sample were collected on a 180 µm × 20 mm 5 µm Symmetry C18 trap column (Waters) flowing at 2500 nL/min and subsequently separated over a 75 µm × 250 mm 1.7 µm Peptide CSH C18 (C18) flowing at 300 nL/min using a nanoAcquity M-Class LC system (Waters). The column was maintained at 60 °C. Solution A was 0.1% formic acid in LC–MS grade water and solution B was 0.1% formic acid in LC–MS grade acetonitrile. The separation gradient was 3 min of isocratic 2.5% B followed by a 7 min gradient to 8% B, then a 30 min gradient to 30% B, a 5-min gradient to 80% B, a 4 min gradient to 95% B, a 1 min gradient to 2.5% B, and 15 min of isocratic 2.5% B. All samples were analysed on an Orbitrap Fusion Tribrid mass spectrometer. TMT-labelled samples were analysed using synchronous precursor selection MS^3^ analysis [63]. MS/MS peaks were picked using Compass. MS2/MS3 spectra were paired using mascot. Searches were performed against the predicted proteome of each fungal species supplemented with common contaminants using Mascot with a mass tolerance of 5 ppm and a false discovery rate of 1%. Variable modifications, including cysteine carbamidomethylation, methionine oxidation, and cysteine, or glutamate modification with 1 was included in the search. TMT ratios were determined using Scaffold. For quantitative analysis, protein hits were filtered for > 2 quantifiable peptide matches at 95% confidence.

### Production and purification of recombinant enzymes in *Pichia pastoris*

Amino acid sequences were selected for recombinant production at random from collections of homologous sequences detected across multiple pulldowns. These included LsGH5_5A, LsGH5_7A, LsGH10A, and TlGH12A (sequences found in Additional file 11: Table S4). Genes, with signal peptides removed [64], were synthesized and cloned into pPICZαA between the EcoRI and SalI restriction sites by Genscript (the Netherlands) to generate sequences with α-factor secretion signals and C-terminal 6×Histidine purification tags. Plasmids were propagated in *E. coli* Stellar cultured in low-salt LB with 25 μg/mL zeocin. For transformation, ~ 1 μg of plasmid DNA was digested with SacI and purified using a PCR purification kit. ~ 100 ng of the resulting linearized DNA was electroporated into *Pichia pastoris* X-33 prepared following the method of Wu et al. [65]. From each transformation, a selection of 3–8 colonies that grew on YPD supplemented with 100 μg/mL of zeocin was streaked for purity. A single colony was taken from each streak plate and grown overnight in 5 mL of BMGY, then induced with two additions of 50 μL (1% final) methanol over 2 days. Culture supernatants were checked for protein of interest via SDS-PAGE and staining with Coomassie dye. The best-producing colony was used for scale-up to 500 mL cultures in 2.5-L baffled flasks, induced in the same manner. Supernatant was collected following centrifugation. The pH was adjusted to 7.5 with NaOH, the cultures were 0.45 μm filtered, and protein was collected on a 5 mL Histrap FF crude column (GE Healthcare). Following a 10 CV wash with 20 mM imidazole, 300 mM NaCl, 20 mM NaP_i_, and pH 7.5, bound protein was eluted with a gradient from 20 to 500 mM imidazole in the same buffer. Protein-bearing elution fractions were pooled, concentrated using a 10 kDa MWCO centrifugal filter, and then purified into 20 mM sodium acetate pH 6, 100 mM NaCl using XK 16/60 columns containing Superdex 75 (TlGH12A) or Superdex 200 (LsGH5_5A, LsGH5_7A, LsGH10A) medium. Protein-bearing fractions were pooled and concentrated to 10–50 mg/mL using a 10 kDa centrifugal filter and stored at − 80 °C. Two LsGH10A elution peaks were observed from Superdex 200; only the later-eluting peak was used, though both showed activity and ran indistinguishably on SDS-PAGE. The total protein yields were 54 mg/L (6xHis tag intact) for LsGH5_5A, 38 mg/L (6×His tag intact) for LsGH5_7A, 26 mg/L for LsGH10A, and 135 mg/L for TlGH12A. Notably, LsGH5_5A and LsGH5_7A produced extremely well (> 200 mg/L based on SDS-PAGE), but the majority of the protein did not bind to a Histrap column, suggesting proteolytic trimming of the C-terminal tag from these enzymes.

### Hydrolysis of substrates by recombinant enzymes

Polysaccharide hydrolysis was measured through the detection of reducing ends using the BCA assay. Briefly, enzyme (< 10 μg/mL) was mixed with substrate in 50 mM pH 4.0 NaOAc buffer with 100 mM NaCl and incubated at 30 °C for 15 min. The reaction was stopped by the addition of freshly mixed BCA reagent (250 mM Na_2_CO_3_, 140 mM NaHCO_3_, 2.5 mM bicinchoninic acid, 1.25 mM CuSO_4_, 2.5 mM l-serine); then colour was developed by incubation at 80 °C for 10 min before measuring A_563_. Reducing ends were determined relative to a glucose calibration series from 10 to 200 μM. A substrate blank was measured and subtracted from each sample measurement. Minor activities were quantified by the same method using 50 μg/mL enzyme with a boiled enzyme control (95 °C, 15 min) added to substrate for background subtraction.

The pH optimum of each enzyme was measured using 1 mg/mL cGM (LsGH5_7A), wAX (LsGH10A), or bMLG (LsGH5_5A, TlGH12A) in a collection of buffers (citrate, acetate, formate, MES, HEPES, phosphate) at different pH values (see Additional file 11: Fig. S15) at 30 °C. The temperature activity profile of each enzyme was measured from 32 to 83 °C using the same substrates in 50 mM pH 4.0 NaOAc buffer. Enzyme was incubated at temperature for 5 min; then substrate was added and reducing ends were quantified relative to a substrate blank following 15 min of incubation with substrate (see Additional file 11: Fig. S16).

Hydrolysis of 4-methylumbelliferyl cellobioside (4MU-GG) and 4-methylumbelliferyl xylobioside (4MU-Xyl2) were quantified at 25 °C in 50 mM pH 4.0 NaOAc buffer using excitation at 360 nm and detection at 450 nm. 4MU fluorescence was calibrated using a dilution series from 100 to 0.8 μM 4MU in the same buffer.

### Inhibition kinetics of recombinant enzymes

Inhibition kinetics were monitored using a continuous assay as described previously [32]. Briefly, enzyme in 100 mM pH 4.0 NaOAc buffer was mixed 1:1, to a final concentration selected to hydrolyse ~ 5% of the substrate over 2 h, with inhibitor and 0.4 mM substrate (diluted from 100 mM in DMSO) in water. Inhibitor concentrations from 0 to 50 μM or 0 to 25 μM were monitored for fluorescence continuously for up to 2 h. To test enzyme recognition specificity, inhibition was measured with glucosyl-β(1,4)-cyclophellitol (GGcyc) [36] or xylosyl-β(1,4)-xylocyclophellitol (XXcyc) [35]. To test the impact of the different linker chemistries, inhibition kinetics were also measured using Biotin-ABP-Xyn [35] and Biotin-ABP-Cel [36].

## Supplementary Information

**Additional file 1.** Proteomic hit information for cellulase pulldown from *A. biennis *secretomes.

**Additional file 2.**  Proteomic hit information for cellulase pulldown from *F. fomentarius* secretomes.

**Additional file 3.** Proteomic hit information for cellulase pulldown from *H. nitida* secretomes.

**Additional file 4. **Proteomic hit information for cellulase pulldown from *L. sp.* 1048 secretomes.

**Additional file 5.** Proteomic hit information for cellulase pulldown from *T. menziesii* secretomes.

**Additional file 6. **Proteomic hit information for cellulase pulldown from *P. brumalis* secretomes.

**Additional file 7. **Proteomic hit information for cellulase pulldown from *P. sanguineus* secretomes.

**Additional file 8. **Proteomic hit information for cellulase pulldown from *T. gibbosa* secretomes.

**Additional file 9. **Proteomic hit information for cellulase pulldown from *T. ljubarskyi* secretomes.

**Additional file 10.** Proteomic hit information for cellulase pulldown from *T. meyenii* secretomes.

**Additional file 11.** Supplementary synthetic methods, figures, and tables.

## Data Availability

*Pichia pastoris* strains and samples of recombinant proteins may be available from Gideon Davies (Gideon.davies@york.ac.uk). Samples of ABP-Cel, ABP-Xyl, and ABP-Glc may be available from Herman Overkleeft (h.s.overkleeft@lic.leidenuniv.nl). Basidiomycete fungi are available from the fungal culture collection of the International Centre of Microbial Resources (CIRM-CF) at the French National Institute for Agricultural research (INRA; Marseille, France). Genome sequences for each of the fungi used in this study are available from Mycocosm (https://mycocosm.jgi.doe.gov/mycocosm/home) (DOE Joint Genome Institute, Walnut Creek, California). Other datasets used and/or analysed during the current study are available from the corresponding author on reasonable request.

## Abbreviations

ABP: Activity-based probe
ABPP: Activity-based protein profiling
BCA: Bicinchoninic acid
bMLG: Barley mixed-linkage glucan
CAZyme: Carbohydrate-active enzyme
cGM: Carob galactomannan
CMC: Carboxymethyl cellulose
DMSO: Dimethylsulfoxide
DTT: Dithiothreitol
GH: Glycoside hydrolases
IAA: Iodoacetamide
LPMO: Lytic polysaccharide monooxygenase
MW: Molecular weight
PVPP: Polyvinylpolypyrrolidone
SDS-PAGE: Sodium dodecyl sulphate-polyacrylamide gel electrophoresis
TEAB: Tetraethylammonium bicarbonate
TMT: Tandem mass tag
wAX: Wheat arabinoxylan
