# Supplementary material for: Activity-based protein profiling reveals dynamic substrate-specific cellulase secretion by saprotrophic basidiomycetes
Source: Biotechnol Biofuels Bioprod. 2022 Jan 17;15:6. doi: 10.1186/s13068-022-02107-z (PMC8764865; doi:10.1186/s13068-022-02107-z)
Supplement: Supplementary file 11 — Additional file 11. Supplementary synthetic methods, figures, and tables. [file 13068_2022_2107_MOESM11_ESM.docx]

# Additional Figures and Tables

# for

**Activity-Based Protein Profiling Reveals Dynamic Substrate-Specific Cellulase Secretion by Saprotrophic Basidiomycetes**

Nicholas G.S. McGregor, ^[a]^ Casper de Boer,^[b]^ Mikhaaeel Santos,^[a]^ Mireille Haon,^[c,d]^ David Navarro,^[c,d]^ Sybrin Schroder,^[b]^ Jean-Guy Berrin,^[c,d]^ Herman S. Overkleeft*^[b]^, Gideon J. Davies,^[a]^

[a] York Structural Biology Laboratory, Department of Chemistry, The University of York, Heslington, York, YO10 5DD

[b] Leiden Institute of Chemistry, Leiden University, Einsteinweg 55, 2300 RA Leiden, The Netherlands

[c] INRA, UMR1163 Biodiversité et Biotechnologie Fongiques, Faculté des Sciences de Luminy, ESIL Polytech, F-13288, Marseille, France

[d] Polytech Marseille, Aix Marseille Université, F-13288, Marseille, France

**Additional Synthetic Protocols and Compound Characterisation**

*t*-Bu-TEG-Cy3 (S2)

Reaction of **S1** (1) (132 mg, 0.50 mmol) with Cy3-carboxylic acid (2) (229 mg, 0.5 mmol) according to general procedure A followed by flash chromatography (DCM/MeOH, 1/0 -> 94.5/5.5 v/v) afforded the title compound as a red solid (0.24 g, 0.33 mmol 66%).

^1^H NMR (300 MHz, CDCl_3_) δ = 8.45 (t, *J*=13.5, 1H), 7.57 – 7.36 (m, 5H), 7.34 – 7.06 (m, 5H), 6.98 (d, *J*=13.4, 1H), 4.17 (t, *J*=7.7, 2H), 4.02 (d, *J*=1.4, 2H), 3.81 (d, *J*=1.5, 3H), 3.76 – 3.55 (m, 10H), 3.46 (dd, *J*=7.8, 3.8, 2H), 2.38 (t, *J*=7.1, 2H), 1.90 (q, *J*=9.3, 8.5, 2H), 1.75 (d, *J*=2.2, 14H), 1.71 – 1.58 (m, 2H), 1.47 (s, 9H). ^13^C NMR (75 MHz, CDCl_3_) δ 174.1, 173.5, 173.3, 150.4, 142.3, 141.5, 140.3, 140.1, 128.6, 128.6, 125.2, 122.0, 121.9, 110.7, 110.5, 104.2, 103.7, 70.3, 70.1, 70.1, 69.8, 69.3, 68.6, 48.8, 48.7, 46.1, 44.4, 38.6, 35.8, 31.9, 27.8, 27.8, 26.8, 26.0, 24.9. HRMS (ESI) m/z: [M]^+^ calculated for C_42_H_60_N_3_O_6_ 702.4477, found 702.4473.

COOH-TEG-Cy3 (S3)

*Tert*-butyl ester **S2** (121 mg, 0.165 mmol) was dissolved in TFA/DCM (2.42 ml, 0.1 M, 17%) and stirred for 4 hours at rt. The mixture was diluted with toluene (20 ml) and evaporated (3x) to furnish the product as a red solid (112 mg, 0.164 mmol, quant.).

^1^H NMR (400 MHz, CDCl_3_) δ = 8.41 (t, *J*=13.4, 1H), 7.83 (t, *J*=5.6, 1H), 7.48 – 7.34 (m, 4H), 7.34 – 7.23 (m, 3H), 7.16 (dd, *J*=8.0, 6.0, 2H), 6.49 (dd, *J*=19.3, 13.5, 2H), 4.20 (s, 2H), 4.06 (t, *J*=7.8, 2H), 3.80 – 3.72 (m, 2H), 3.73 – 3.55 (m, 11H), 3.52 – 3.43 (m, 2H), 2.42 (t, *J*=7.5, 2H), 1.89 – 1.64 (m, 16H), 1.60 – 1.49 (m, 2H). ^13^C NMR (101 MHz, CDCl_3_) δ 176.0, 174.6, 174.2, 172.4, 150.6, 142.6, 141.8, 140.6, 140.4, 129.2, 129.0, 125.8, 125.7, 122.3, 122.2, 111.2, 110.9, 103.6, 103.4, 71.1, 70.5, 70.4, 70.0, 69.4, 68.9, 49.4, 49.2, 46.3, 44.5, 39.8, 35.5, 31.5, 28.1, 28.1, 27.1, 26.3, 25.6. HRMS (ESI) m/z: [M]^+^ calculated for C_38_H_52_N_3_O_6_ 646.38506 found 646.38514.

ABP-Cel-Cy3^+^

**S3** (25 μmol) was dissolved in DMF (0.5 ml), 2,3,4,5,6-pentafluorophenol (23 mg, 0.13 μmol), Et_3_N (10 μl, 0.13 mmol) and DIC (3.9 μl,25 μmol) were added and the mixture was stirred for 90 minutes. Part of the stock solution (0.34 ml) was added to **S4** (1) (4.7 mg, 14 μmol) and stirred overnight. LC-MS indicated full conversion and the product was purified on semi-preparative HPLC eluting with a linear gradient of solution A (MeCN) in solution B (50 mM AcOH in H_2_O). The fractions were concentrated under reduced pressure, co-evaporated with water, diluted with water and lyophilized to yield the product as a red solid (9.7 mg, 9.7 μmol, 69%).

^1^H NMR (600 MHz, D_2_O) δ = 8.48 (t, *J*=13.4, 1H), 7.59 – 7.53 (m, 2H), 7.50 – 7.43 (m, 2H), 7.37 – 7.30 (m, 4H), 6.36 – 6.26 (m, 2H), 4.46 (d, *J*=8.0, 1H), 4.13 – 4.05 (m, 5H), 3.95 – 3.88 (m, 1H), 3.87 – 3.80 (m, 2H), 3.73 – 3.65 (m, 6H), 3.66 – 3.54 (m, 10H), 3.54 – 3.47 (m, 4H), 3.42 – 3.37 (m, 1H), 3.31 – 3.26 (m, 2H), 3.23 (d, *J*=3.8, 1H), 2.33 – 2.27 (m, 1H), 2.28 – 2.22 (m, 2H), 1.90 – 1.82 (m, 2H), 1.73 – 1.70 (m, 12H), 1.69 – 1.62 (m, 2H), 1.41 – 1.32 (m, 2H). ^13^C NMR (151 MHz, D_2_O) δ 177.2, 176.0, 175.5, 173.6, 151.3, 143.3, 142.7, 141.6, 141.5, 129.3, 126.0, 123.0, 122.9, 112.0, 111.7, 103.6, 102.8, 102.7, 78.5, 75.4, 74.4, 73.7, 71.5, 70.8, 70.2, 70.1, 70.0, 69.4, 61.3, 60.5, 57.2, 55.9, 51.7, 49.8, 49.7, 44.3, 43.3, 31.5, 27.9, 27.7, 27.2, 26.0, 25.6. HRMS (ESI) m/z: [M]^+^ calculated for C_51_H_73_N_4_O_14_ 965.5118 found 965.5116.

*t*-Bu-TEG-Cy3 (S2) in CDCl_3_

COOH-TEG-Cy3 (S3) in CDCl_3_

ABP Cel Cy3 in D_2_O

**Additional Figures**


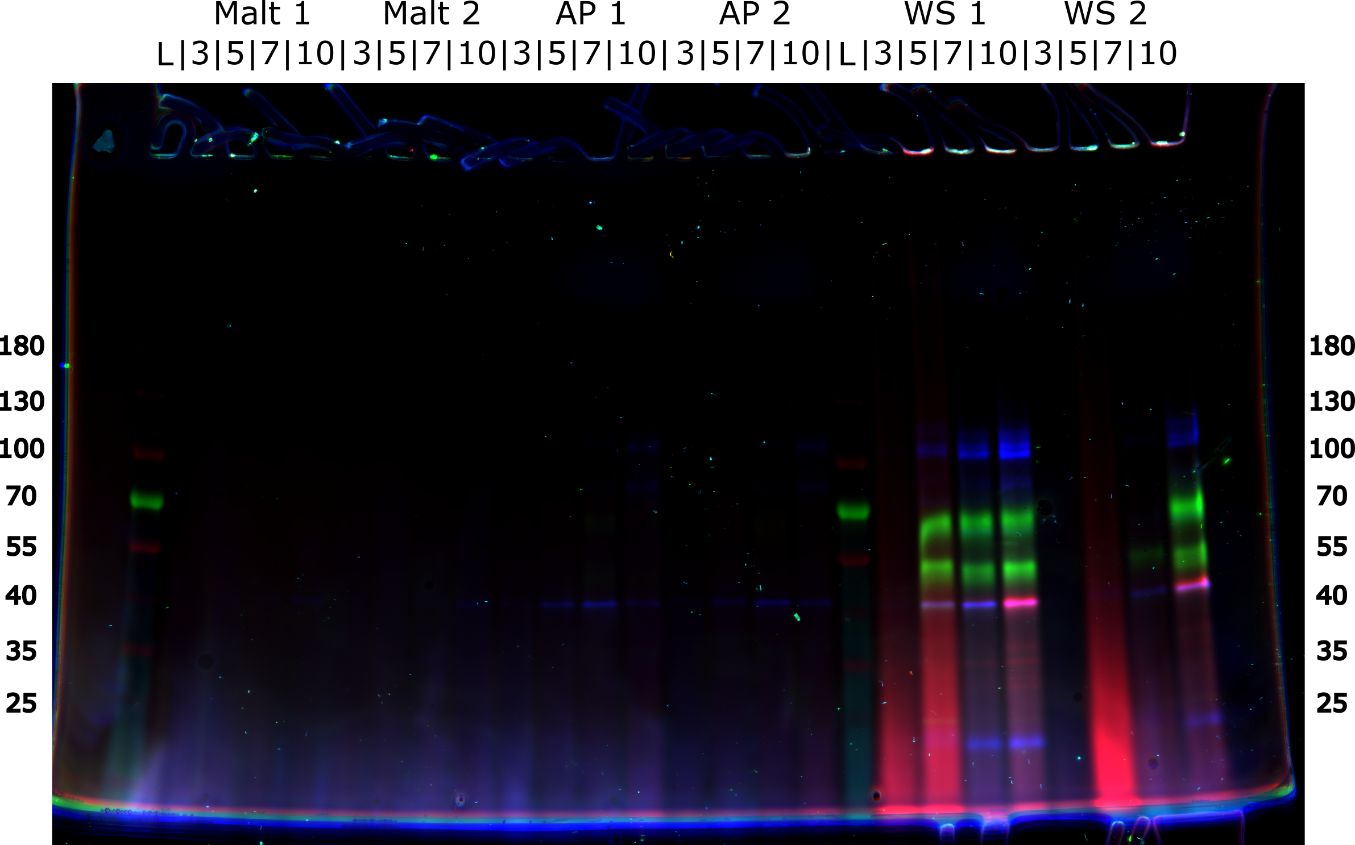


Figure S1: SDS-PAGE of *T. gibbosa* secretomes. Multiplex fluorescence scans using the Cy2, Cy3, and Cy5 laser/filter sets of 4-15% SDS-PAGE-separated basidiomycete secretomes treated with JJB376 (blue, beta-glucosidase), CB644 (green, cellulase), and SYF230 (red, xylanase). Labels are shown above each lane. L indicates ladder, AP indicates secretome grown on aspen pulp, WS indicates secretome grown on wheat straw, Malt indicates secretome grown on maltose, the number of above each lane indicates the day at which the sample was collected.


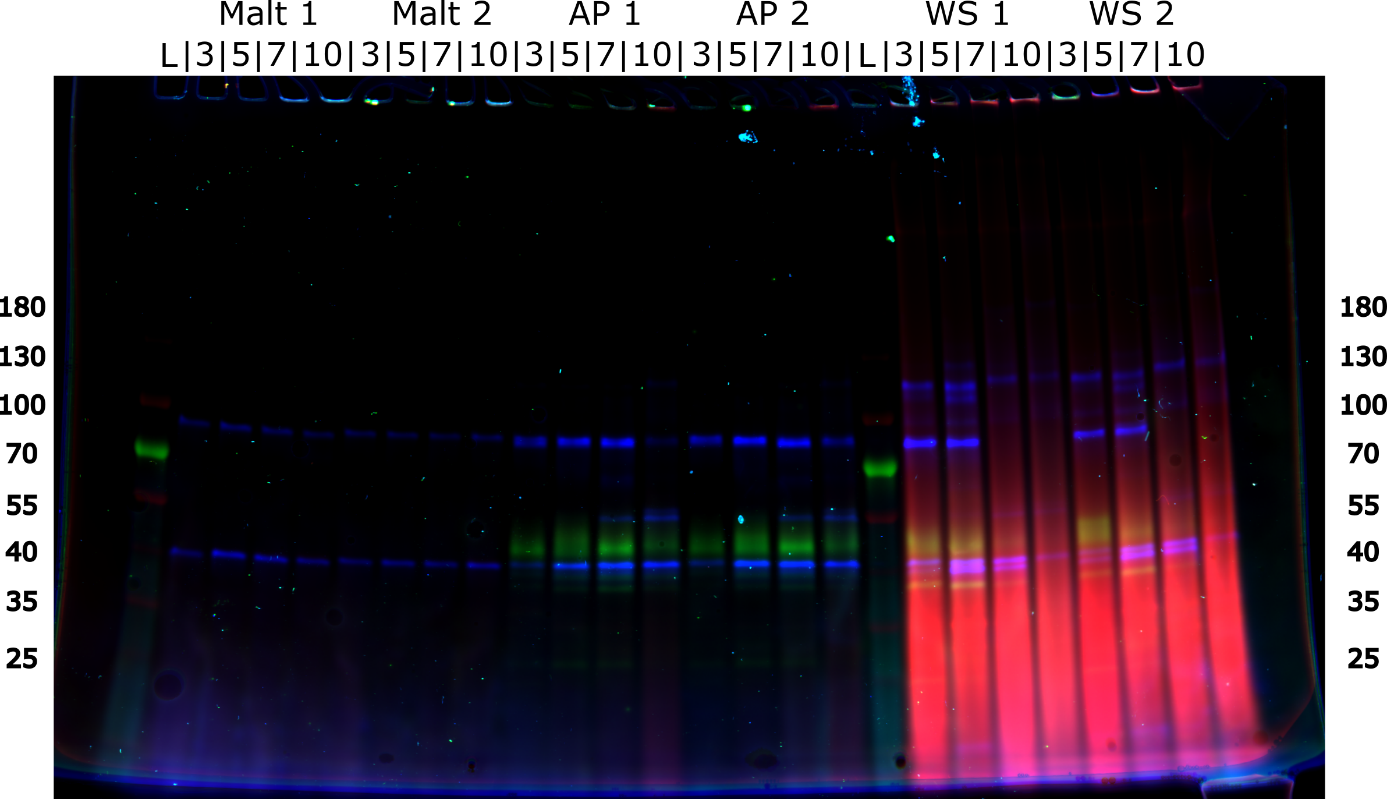


Figure S2: SDS-PAGE of *P. brumalis* secretomes. Multiplex fluorescence scans using the Cy2, Cy3, and Cy5 laser/filter sets of 4-15% SDS-PAGE-separated basidiomycete secretomes treated with JJB376 (blue, beta-glucosidase), CB644 (green, cellulase), and SYF230 (red, xylanase). Labels are shown above each lane. L indicates ladder, AP indicates secretome grown on aspen pulp, WS indicates secretome grown on wheat straw, Malt indicates secretome grown on maltose, the number of above each lane indicates the day at which the sample was collected.


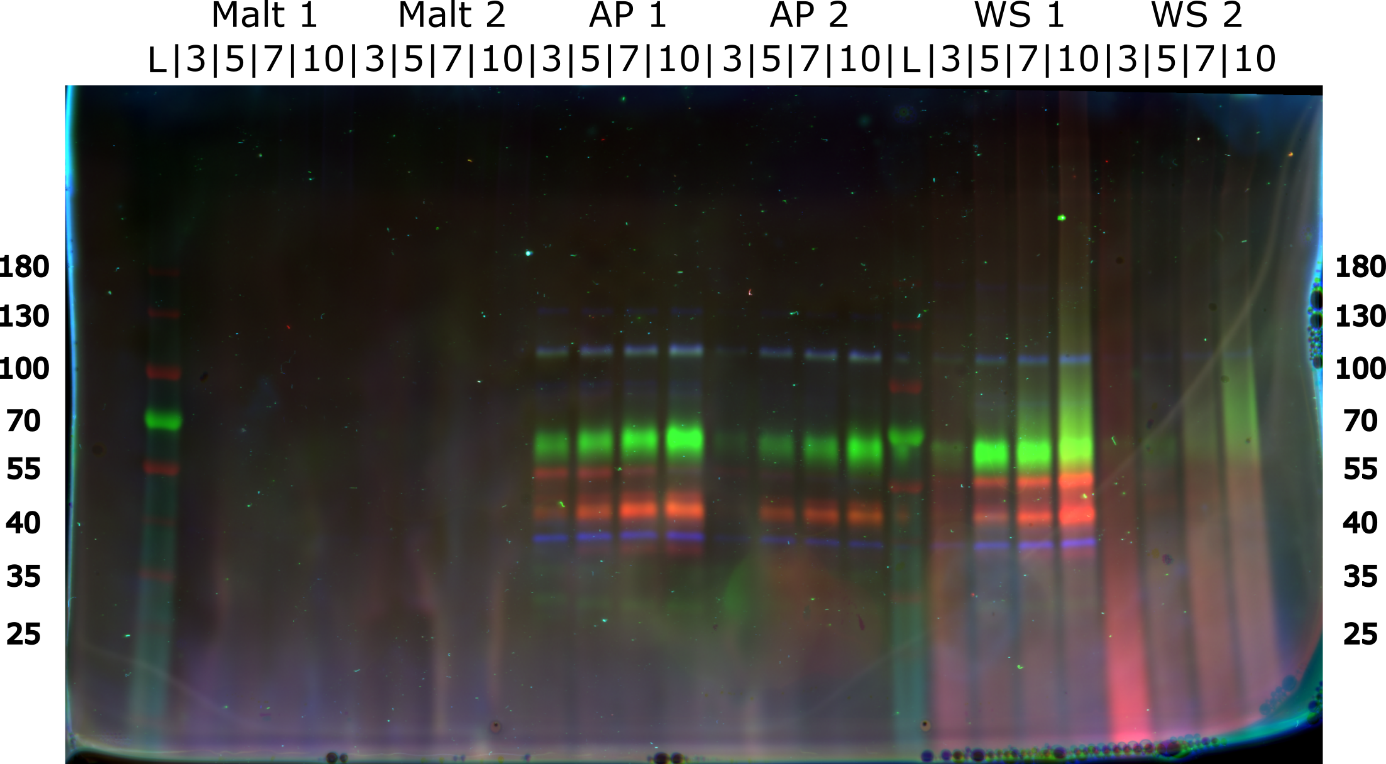


Figure S3: SDS-PAGE of *A. biennis* secretomes. Multiplex fluorescence scans using the Cy2, Cy3, and Cy5 laser/filter sets of 4-15% SDS-PAGE-separated basidiomycete secretomes treated with JJB376 (blue, beta-glucosidase), CB644 (green, cellulase), and SYF230 (red, xylanase). Labels are shown above each lane. L indicates ladder, AP indicates secretome grown on aspen pulp, WS indicates secretome grown on wheat straw, Malt indicates secretome grown on maltose, the number of above each lane indicates the day at which the sample was collected.


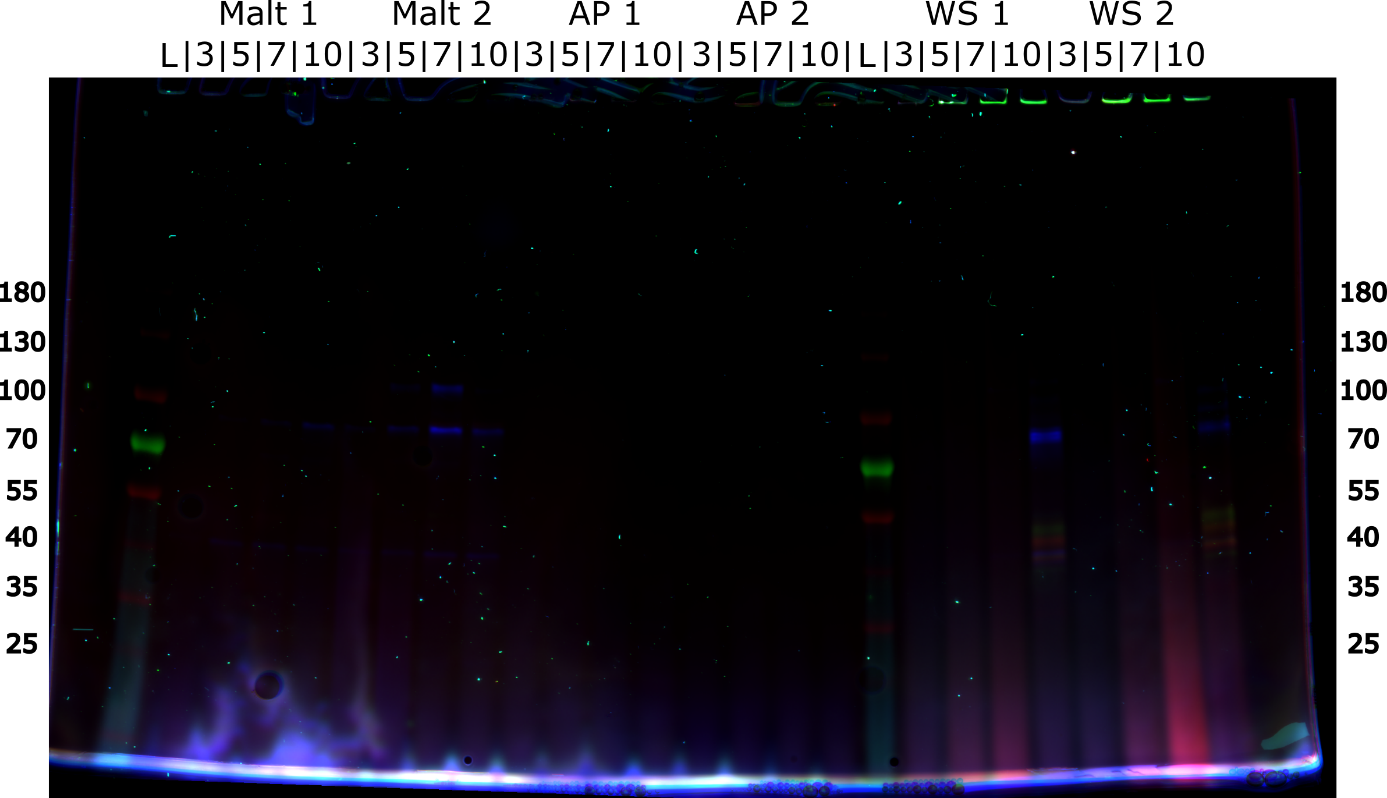


Figure S4: SDS-PAGE of *H. nitida* secretomes. Multiplex fluorescence scans using the Cy2, Cy3, and Cy5 laser/filter sets of 4-15% SDS-PAGE-separated basidiomycete secretomes treated with JJB376 (blue, beta-glucosidase), CB644 (green, cellulase), and SYF230 (red, xylanase). Labels are shown above each lane. L indicates ladder, AP indicates secretome grown on aspen pulp, WS indicates secretome grown on wheat straw, Malt indicates secretome grown on maltose, the number of above each lane indicates the day at which the sample was collected.


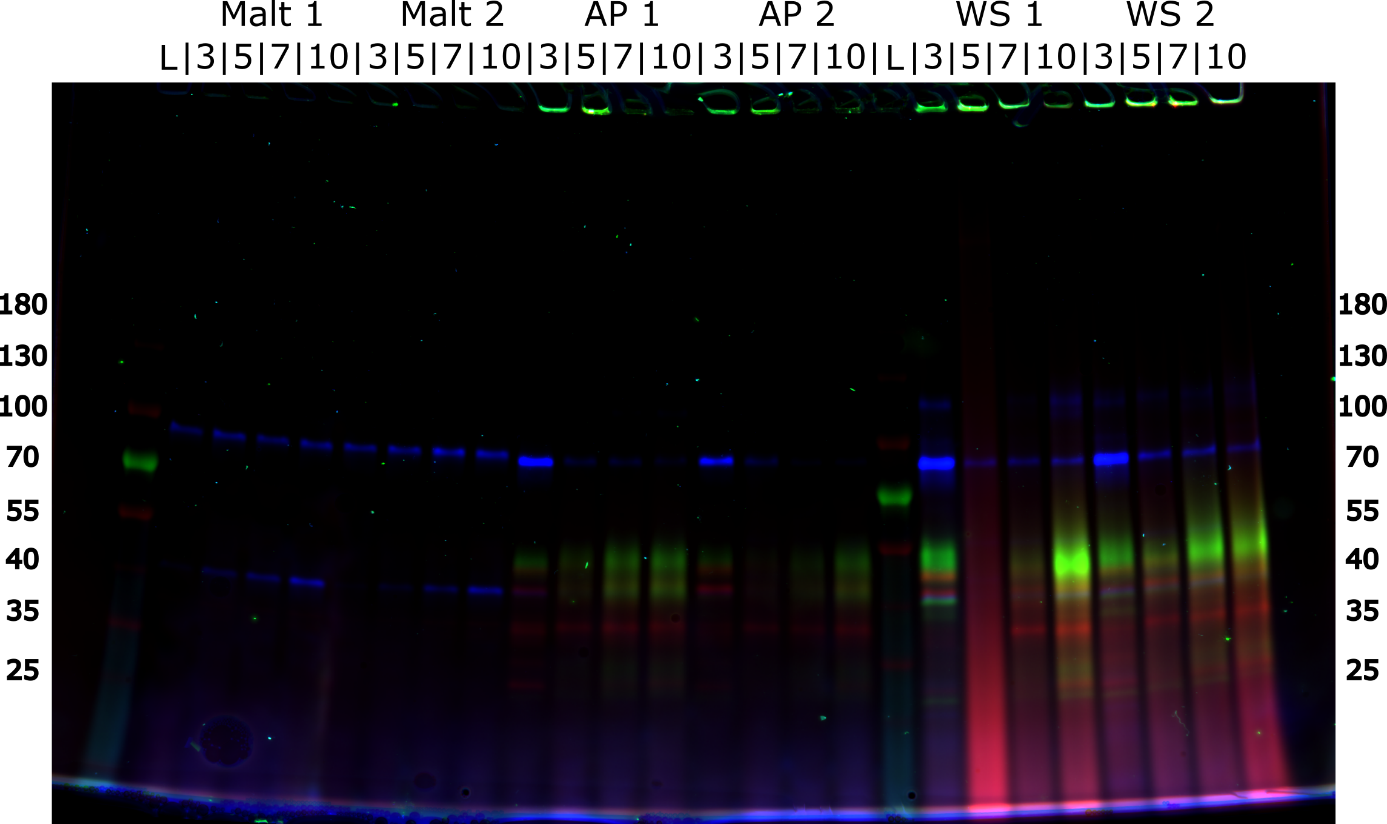


Figure S5: SDS-PAGE of *T. ljubarkyi* secretomes. Multiplex fluorescence scans using the Cy2, Cy3, and Cy5 laser/filter sets of 4-15% SDS-PAGE-separated basidiomycete secretomes treated with JJB376 (blue, beta-glucosidase), CB644 (green, cellulase), and SYF230 (red, xylanase). Labels are shown above each lane. L indicates ladder, AP indicates secretome grown on aspen pulp, WS indicates secretome grown on wheat straw, Malt indicates secretome grown on maltose, the number of above each lane indicates the day at which the sample was collected.


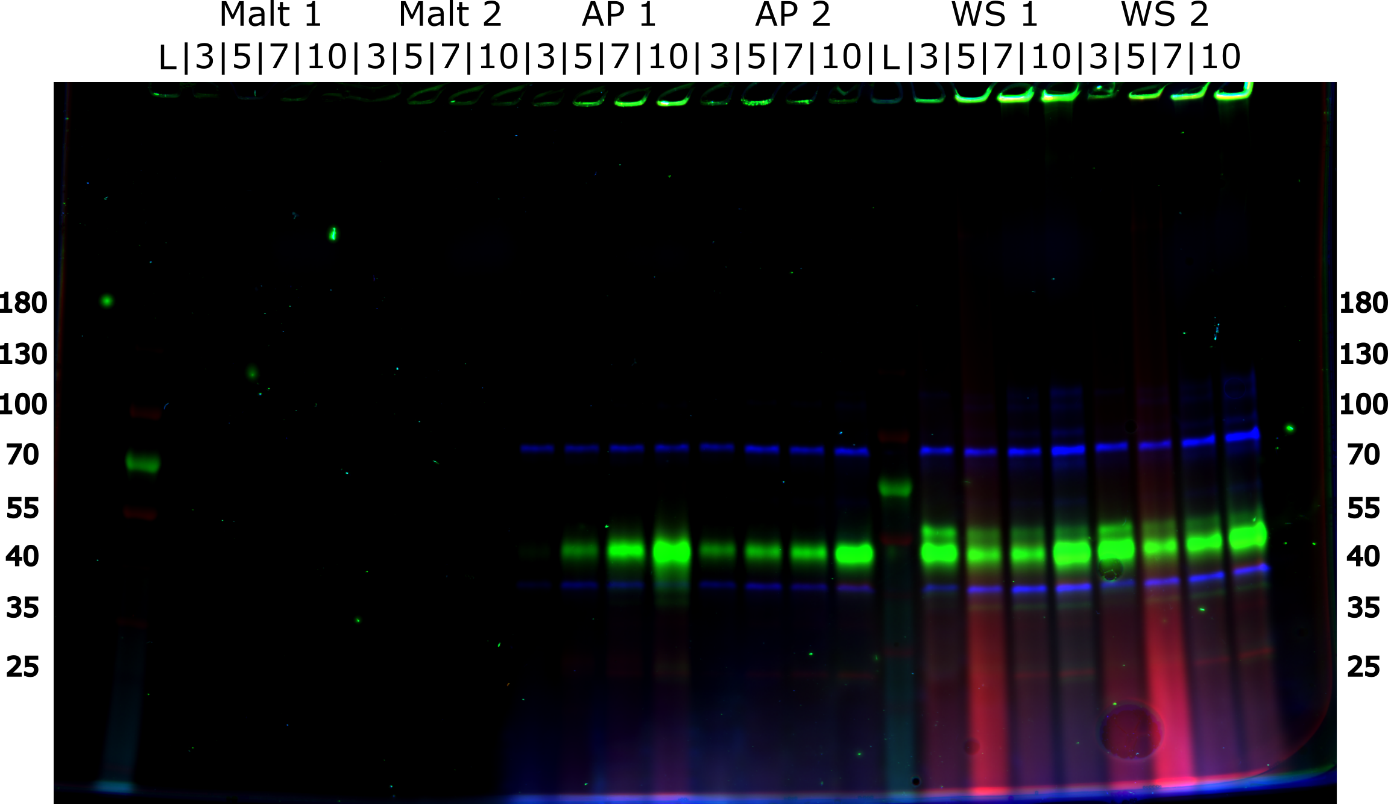


Figure S6: SDS-PAGE of *L. menziesii* secretomes. Multiplex fluorescence scans using the Cy2, Cy3, and Cy5 laser/filter sets of 4-15% SDS-PAGE-separated basidiomycete secretomes treated with JJB376 (blue, beta-glucosidase), CB644 (green, cellulase), and SYF230 (red, xylanase). Labels are shown above each lane. L indicates ladder, AP indicates secretome grown on aspen pulp, WS indicates secretome grown on wheat straw, Malt indicates secretome grown on maltose, the number of above each lane indicates the day at which the sample was collected.


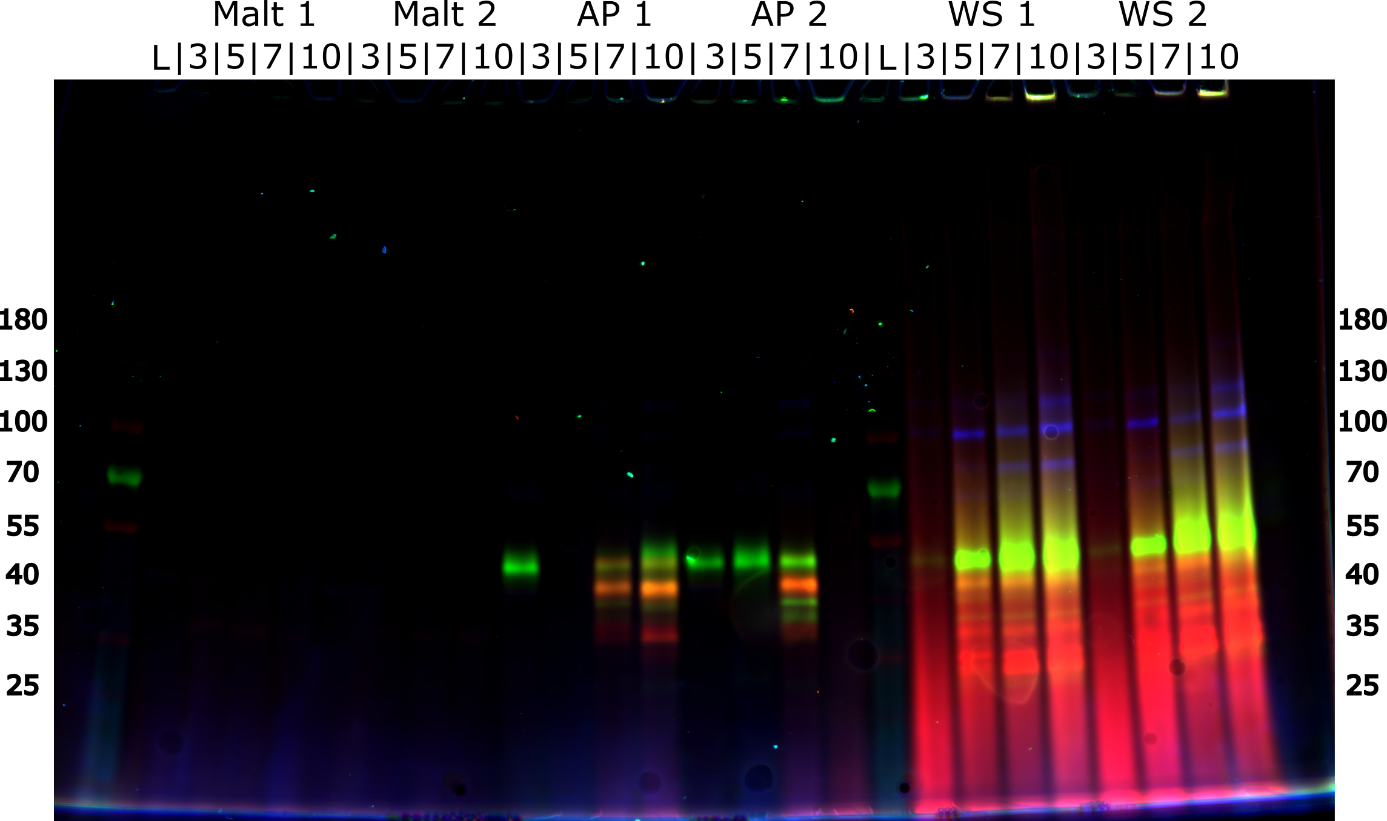


Figure S7: SDS-PAGE of *F. fomentarius* secretomes. Multiplex fluorescence scans using the Cy2, Cy3, and Cy5 laser/filter sets of 4-15% SDS-PAGE-separated basidiomycete secretomes treated with JJB376 (blue, beta-glucosidase), CB644 (green, cellulase), and SYF230 (red, xylanase). Labels are shown above each lane. L indicates ladder, AP indicates secretome grown on aspen pulp, WS indicates secretome grown on wheat straw, Malt indicates secretome grown on maltose, the number of above each lane indicates the day at which the sample was collected.


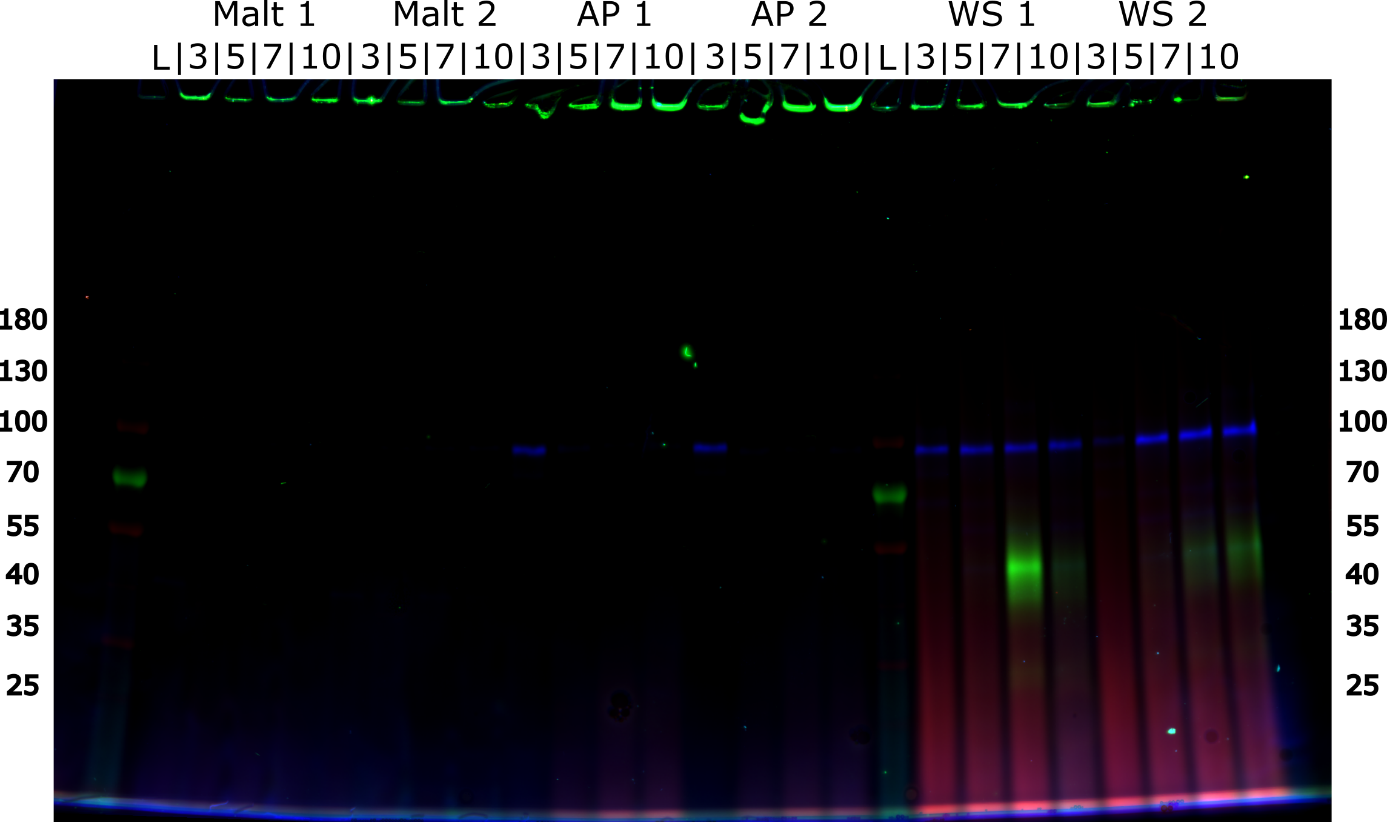


Figure S8: SDS-PAGE of *T. meyenii* secretomes. Multiplex fluorescence scans using the Cy2, Cy3, and Cy5 laser/filter sets of 4-15% SDS-PAGE-separated basidiomycete secretomes treated with JJB376 (blue, beta-glucosidase), CB644 (green, cellulase), and SYF230 (red, xylanase). Labels are shown above each lane. L indicates ladder, AP indicates secretome grown on aspen pulp, WS indicates secretome grown on wheat straw, Malt indicates secretome grown on maltose, the number of above each lane indicates the day at which the sample was collected.


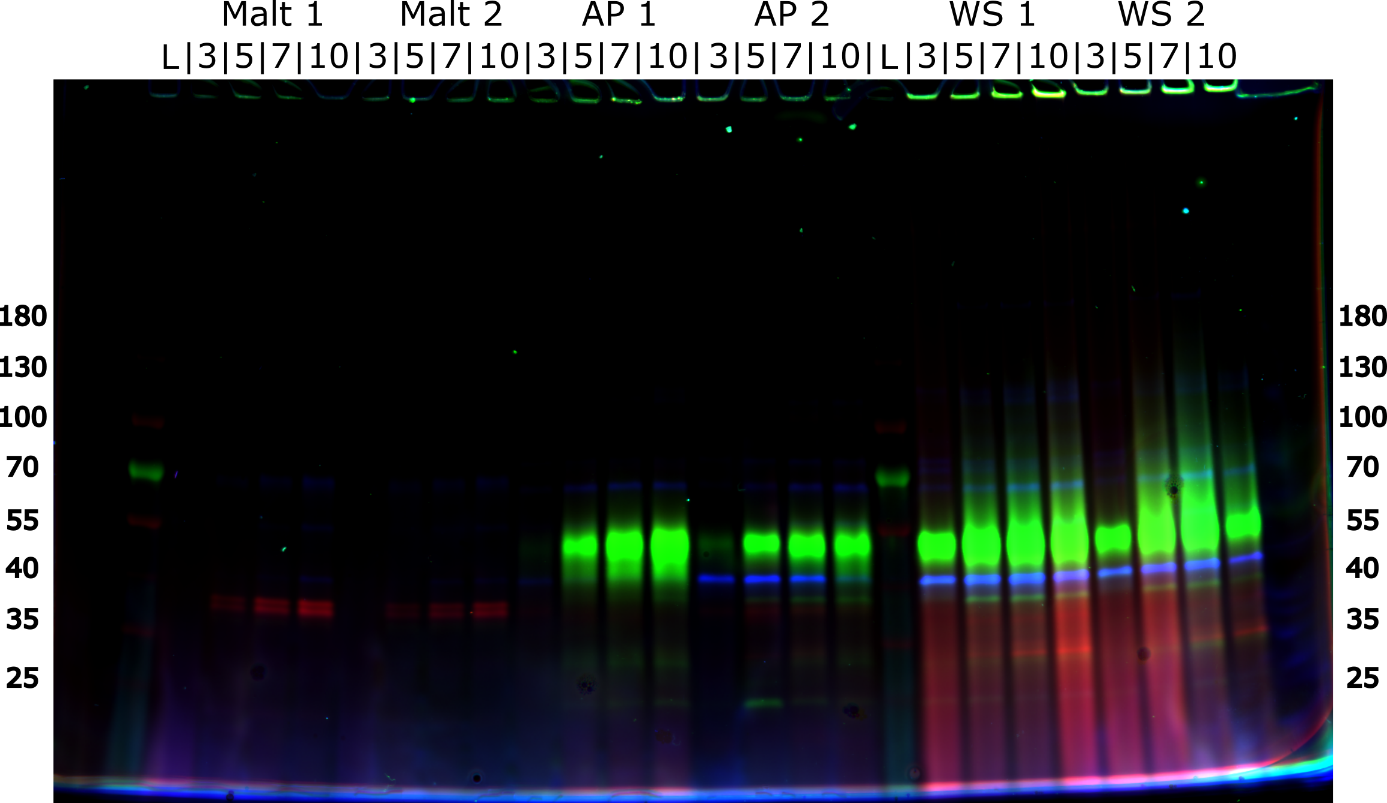


Figure S9: SDS-PAGE of *P. sanguineus* secretomes. Multiplex fluorescence scans using the Cy2, Cy3, and Cy5 laser/filter sets of 4-15% SDS-PAGE-separated basidiomycete secretomes treated with JJB376 (blue, beta-glucosidase), CB644 (green, cellulase), and SYF230 (red, xylanase). Labels are shown above each lane. L indicates ladder, AP indicates secretome grown on aspen pulp, WS indicates secretome grown on wheat straw, Malt indicates secretome grown on maltose, the number of above each lane indicates the day at which the sample was collected.


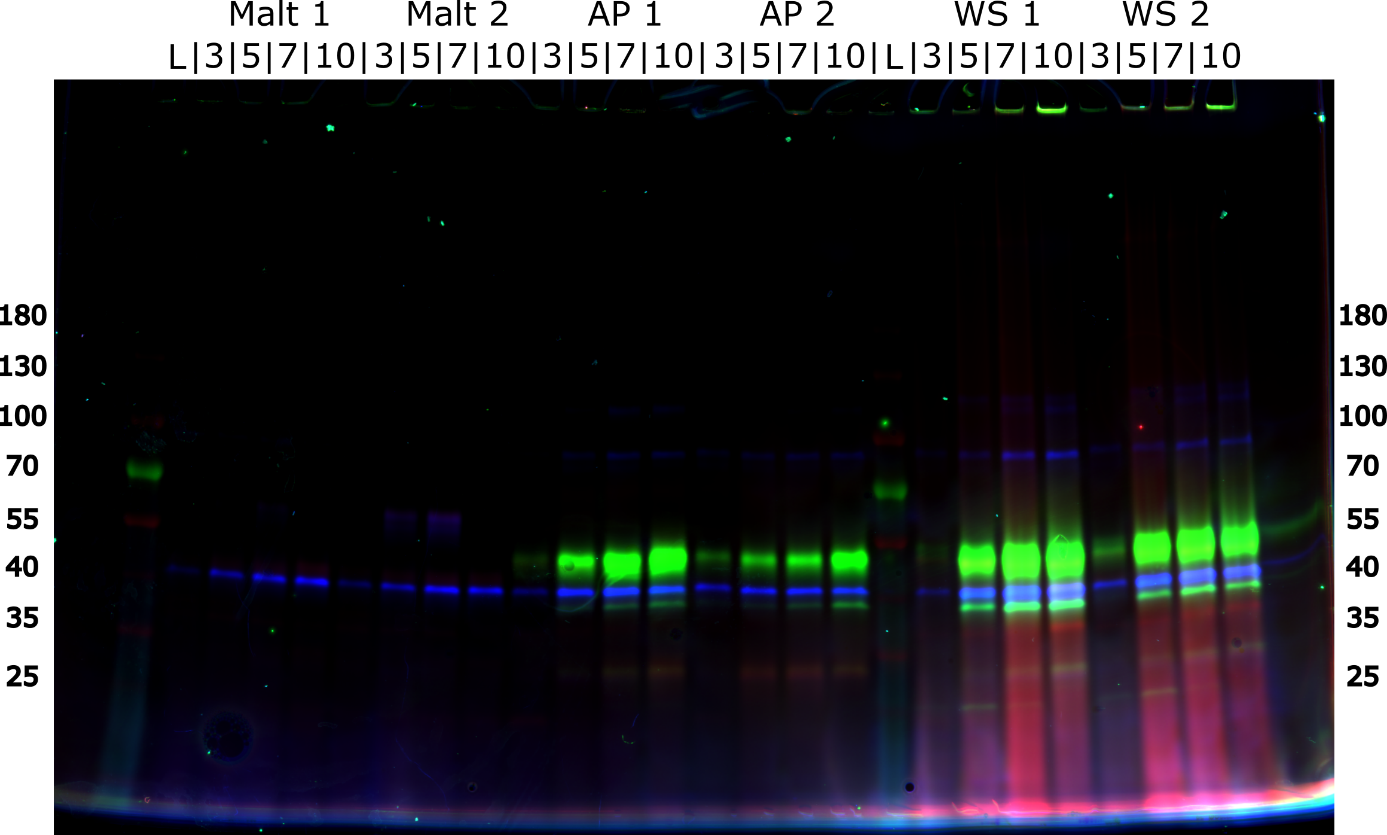


Figure S10: SDS-PAGE of *L. sp.* secretomes. Multiplex fluorescence scans using the Cy2, Cy3, and Cy5 laser/filter sets of 4-15% SDS-PAGE-separated basidiomycete secretomes treated with JJB376 (blue, beta-glucosidase), CB644 (green, cellulase), and SYF230 (red, xylanase). Labels are shown above each lane. L indicates ladder, AP indicates secretome grown on aspen pulp, WS indicates secretome grown on wheat straw, Malt indicates secretome grown on maltose, the number of above each lane indicates the day at which the sample was collected.


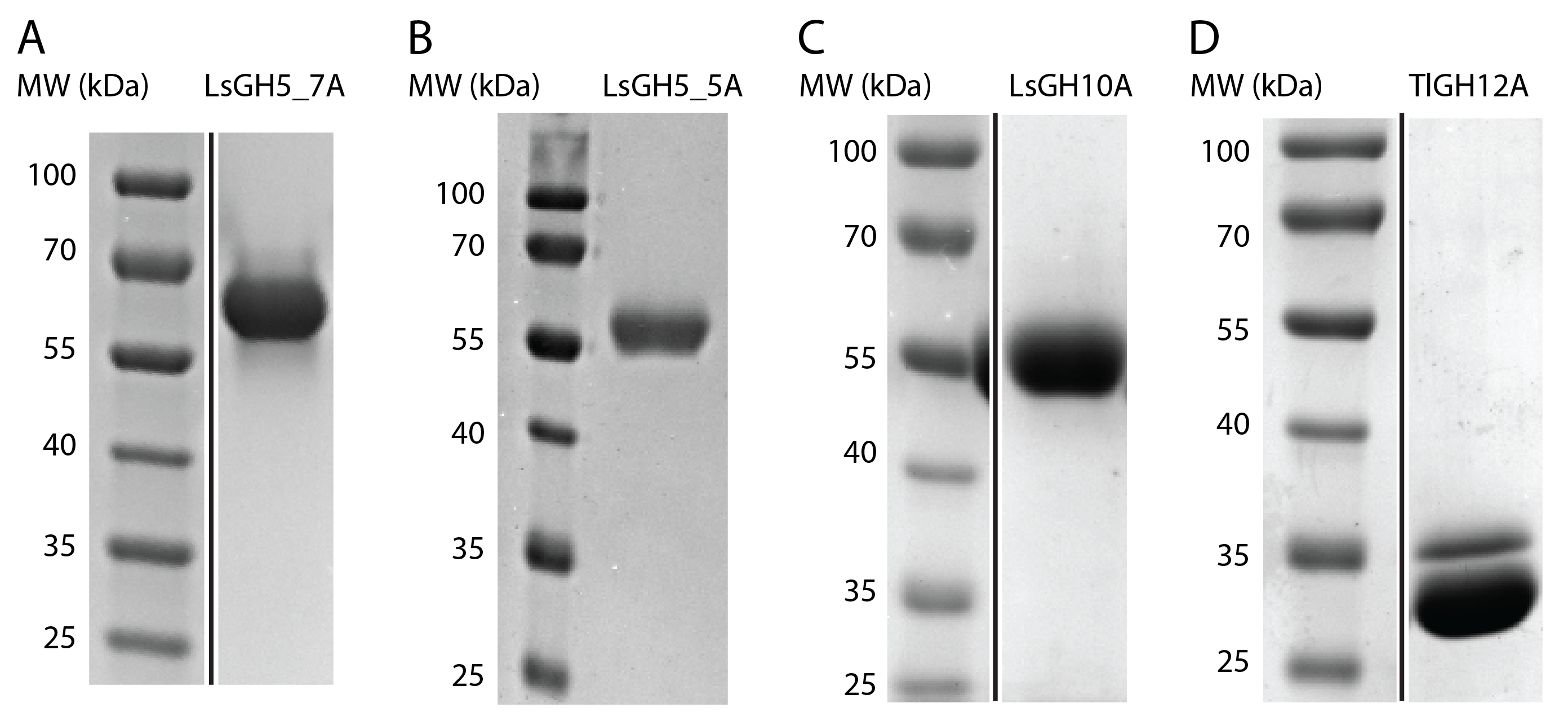


Figure S11: SDS-PAGE of purified recombinant A) LsGH5_7A, B) LsGH5_5A, C) LsGH10A, and D) TlGH12A. The ladder is Thermo Scientific PageRuler Prestained Protein Ladder in all cases. Protein was visualized with Coomassie dye.


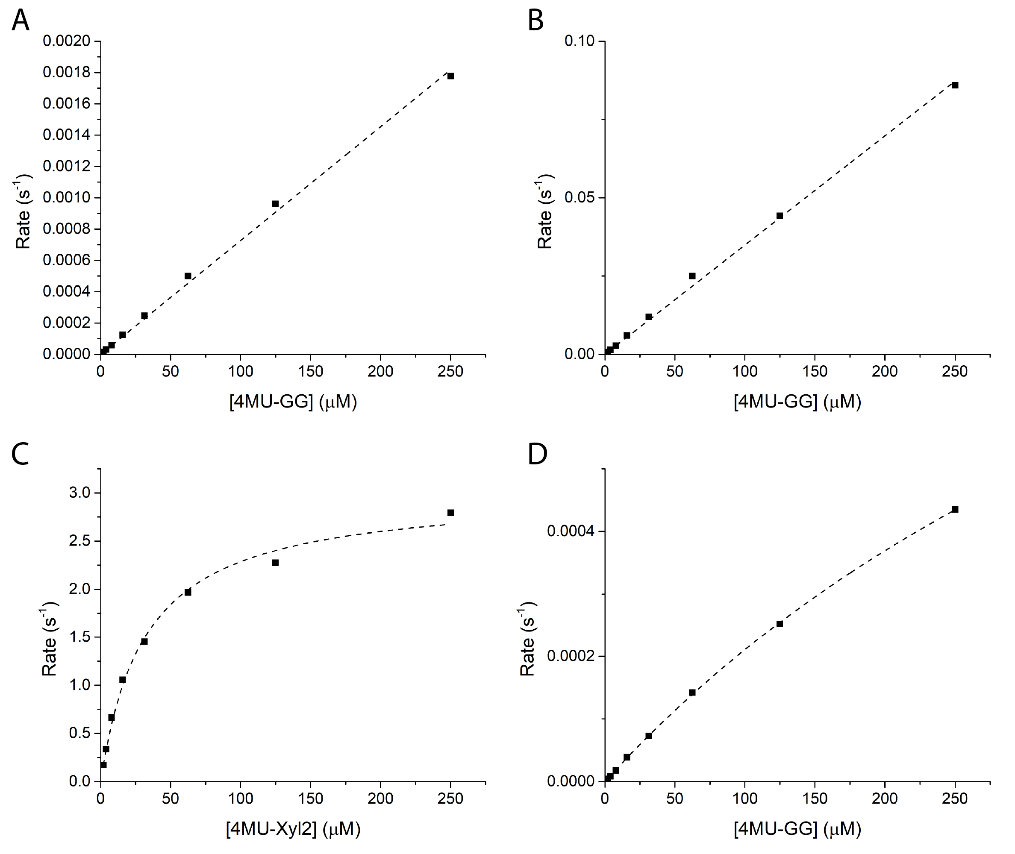
Figure S12: Activity vs. substrate concentration measured for A) LsGH5_5A acting on 4MU-GG B) LsGH10A acting on 4MU-GG C) LsGH10A acting on 4MU-Xyl2 D) TlGH12A acting on 4MU-GG


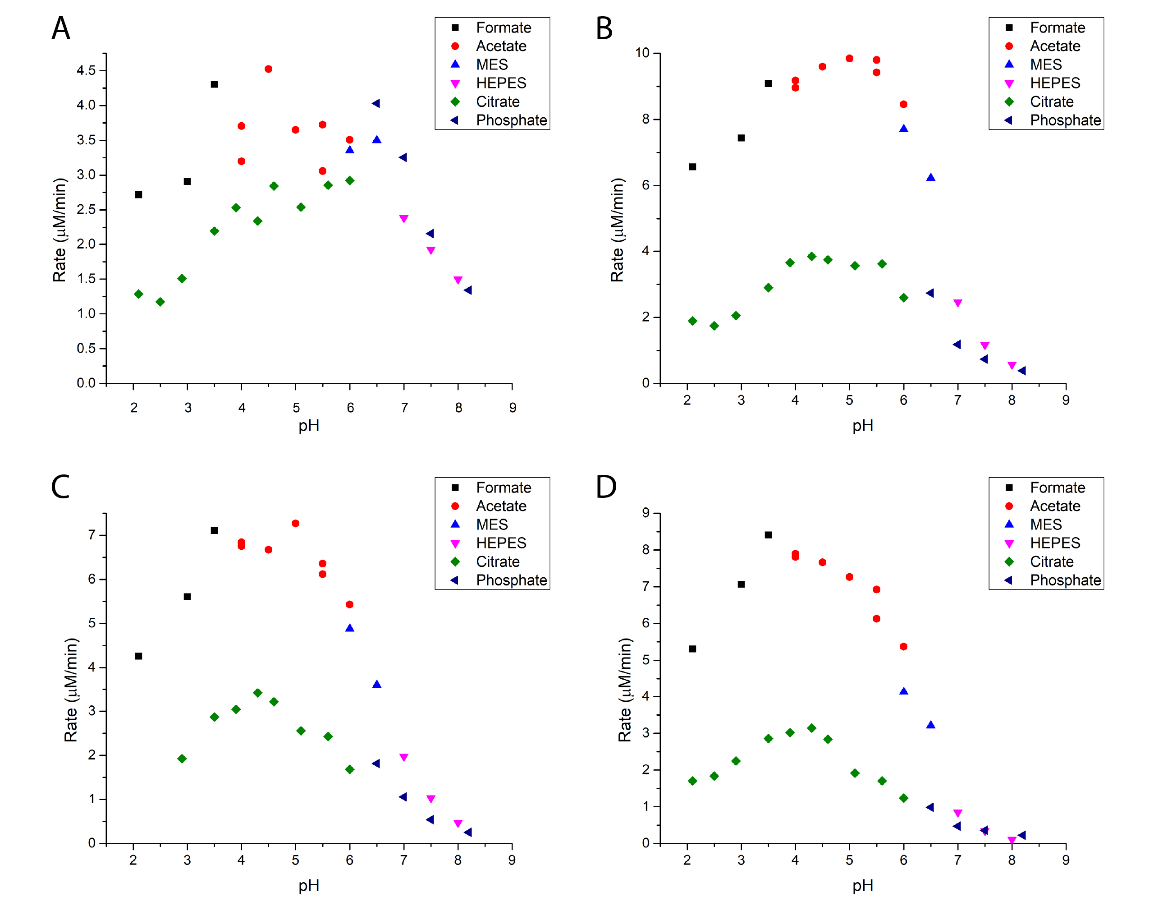
Figure S13: pH-activity profiles measured for A) LsGH5_7A B) LsGH5_5A C) LsGH10A D) TlGH12A


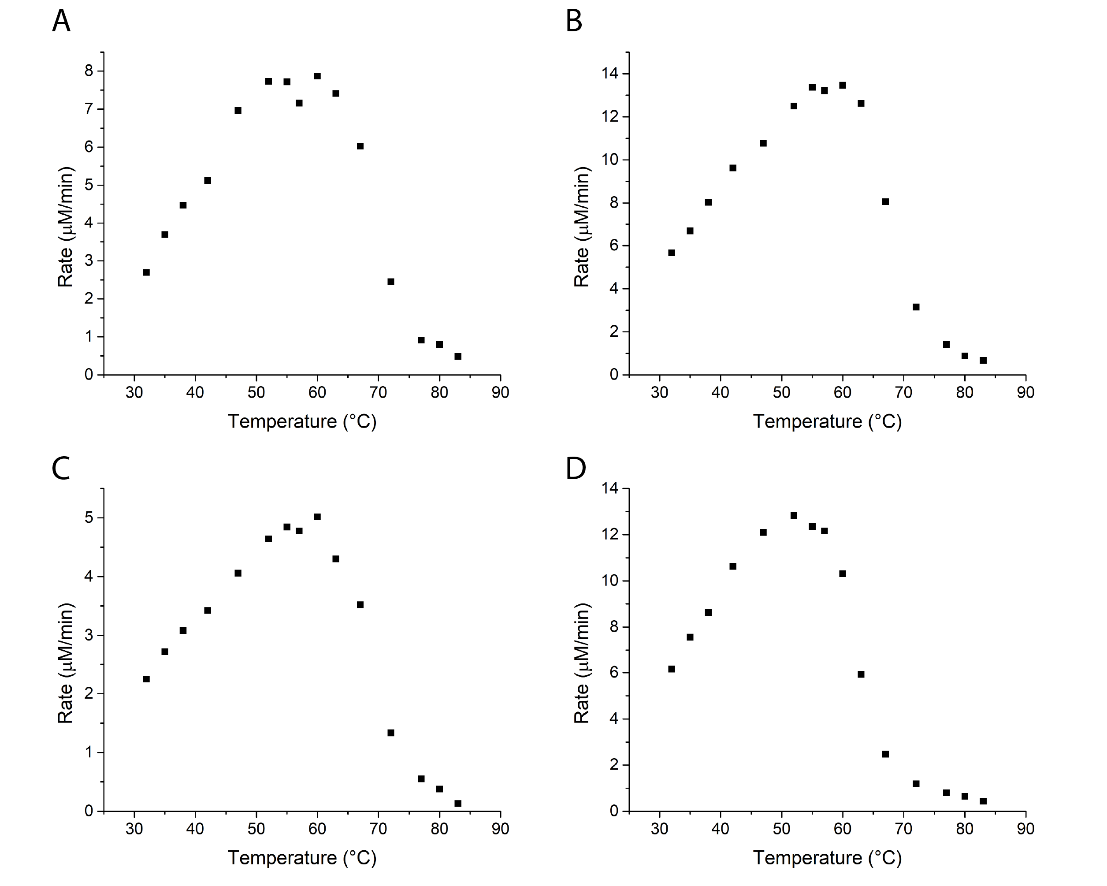


Figure S14: Temperature-activity profiles measured for A) LsGH5_7A B) LsGH5_5A C) LsGH10A D) TlGH12A


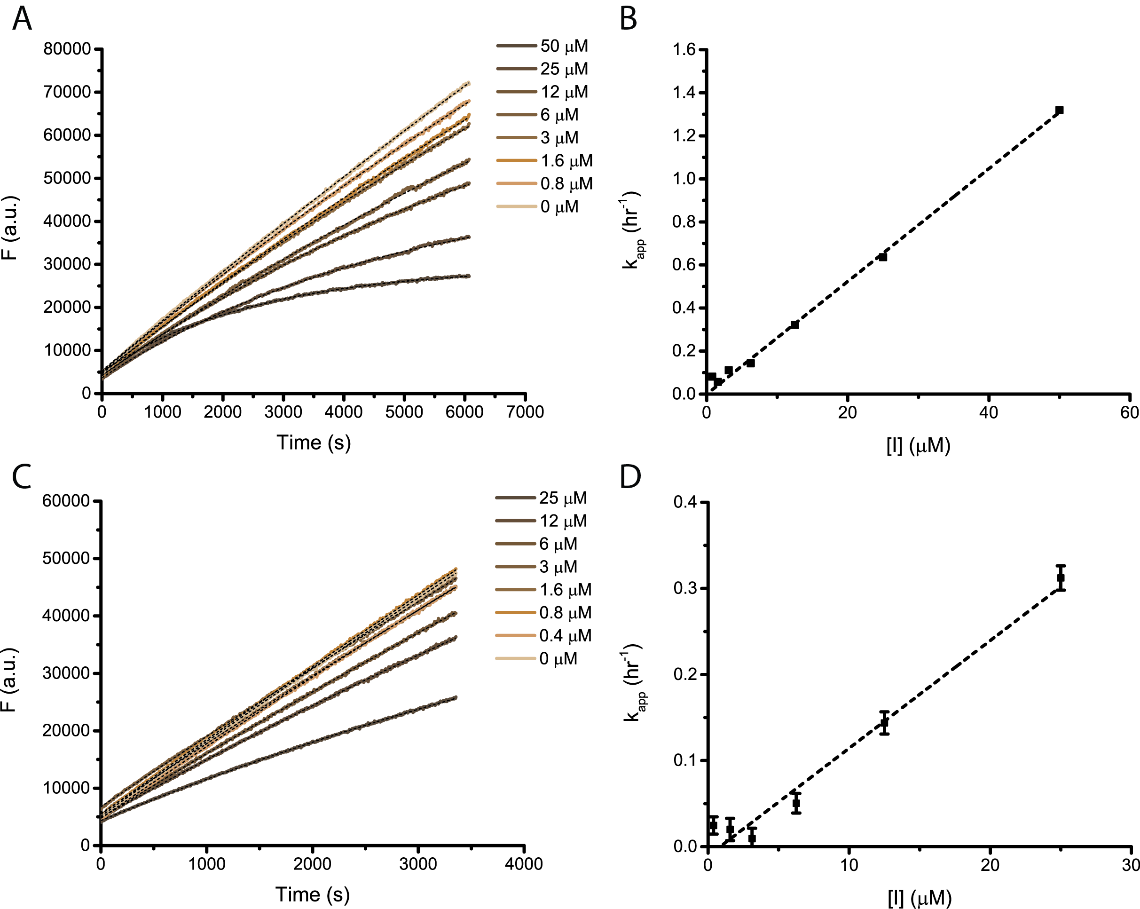


Figure S15: LsGH5_5A inhibition kinetics. A) Fluorescence over time for LsGH5_5A acting on 4MU-GG in the presence of variable concentrations of GGcyc. Exponential decay fit lines for each data set are shown as dashed black lines. B) Apparent decay constant (*k*_app_) vs. inhibitor concentration for LsGH5_5A inhibited by GGcyc. The linear fit used to calculate *k*_inact_/*K*_I_ is shown as a black dash line. Error bars represent the standard error in the *k*_app_ value determined from the fit of the line in A. C) Fluorescence over time for LsGH5_5A acting on 4MU-GG in the presence of variable concentrations of Biotin-ABP-Cel. Exponential decay fit lines for each data set are shown as dashed black lines. D) Apparent decay constant (*k*_app_) vs. inhibitor concentration for LsGH5_5A inhibited by Biotin-ABP-Cel. The linear fit used to calculate *k*_inact_/*K*_I_ is shown as a black dash line. Error bars represent the standard error in the *k*_app_ value determined from the fit of the line in C.


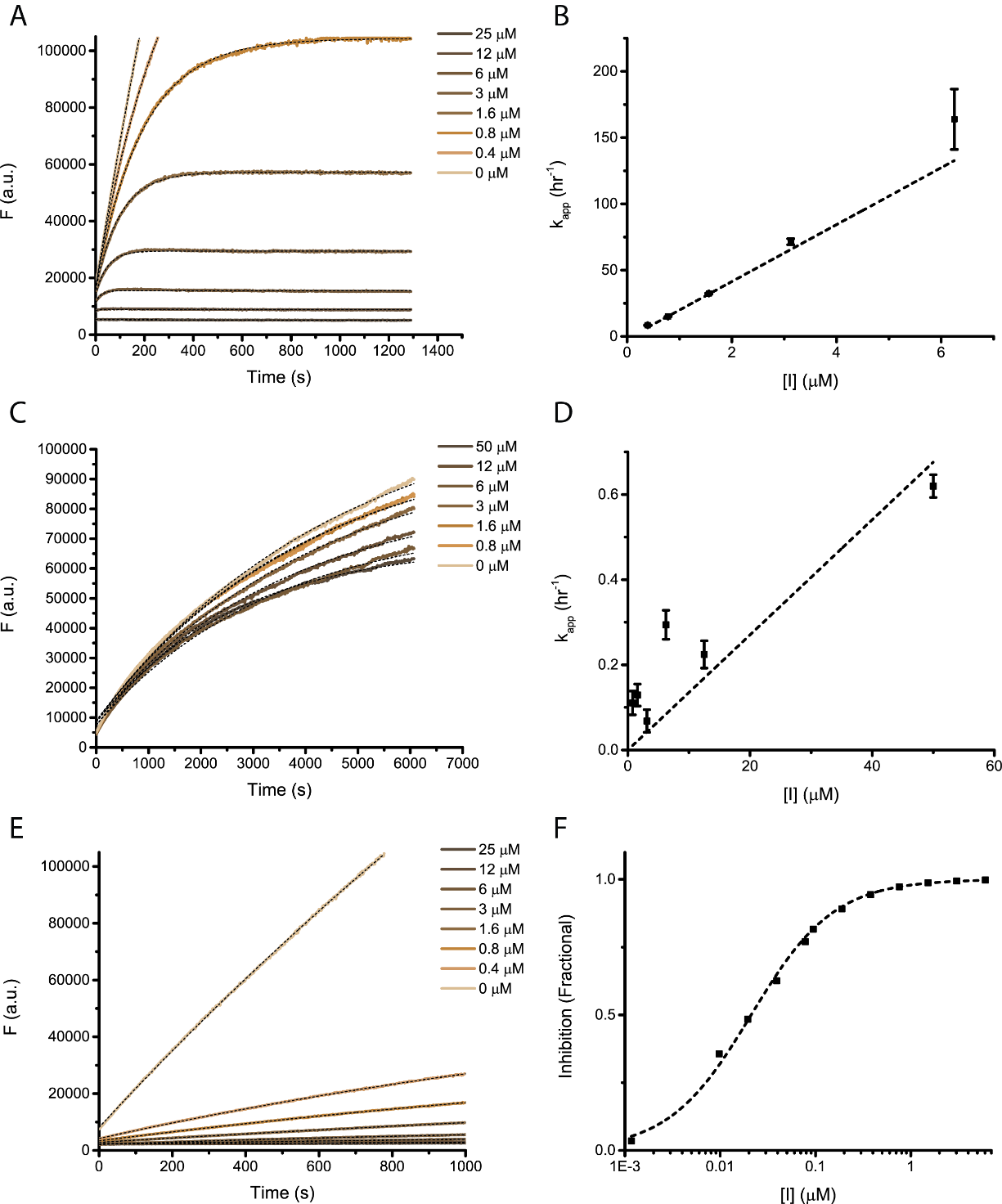


Figure S16: LsGH10A inhibition kinetics. A) Fluorescence over time for LsGH10A acting on 4MU-Xyl2 in the presence of variable concentrations of XXcyc. Exponential decay fit lines for each data set are shown as dashed black lines. B) Apparent decay constant (*k*_app_) vs. inhibitor concentration for LsGH10A inhibited by XXcyc. The linear fit used to calculate *k*_inact_/*K*_I_ is shown as a black dash line. Error bars represent the standard error in the *k*_app_ value determined from the fit of the line in A. C) Fluorescence over time for LsGH10A acting on 4MU-GG in the presence of variable concentrations of GGcyc shown as in panel A. D) Apparent decay constant (*k*_app_) vs. inhibitor concentration for LsGH10A inhibited by Biotin-ABP-Cel, shown as in panel B. E) Fluorescence over time for LsGH10A acting on 4MU-Xyl2 in the presence of variable concentrations of Biotin-ABP-Xyn shown as in panel A. F) No apparent decay was detected, so fractional inhibition (1-v_uninhibited_/v_inhibited_) is shown with a single site saturation kinetic fit used to determine *K*_I_ (black dashed line).


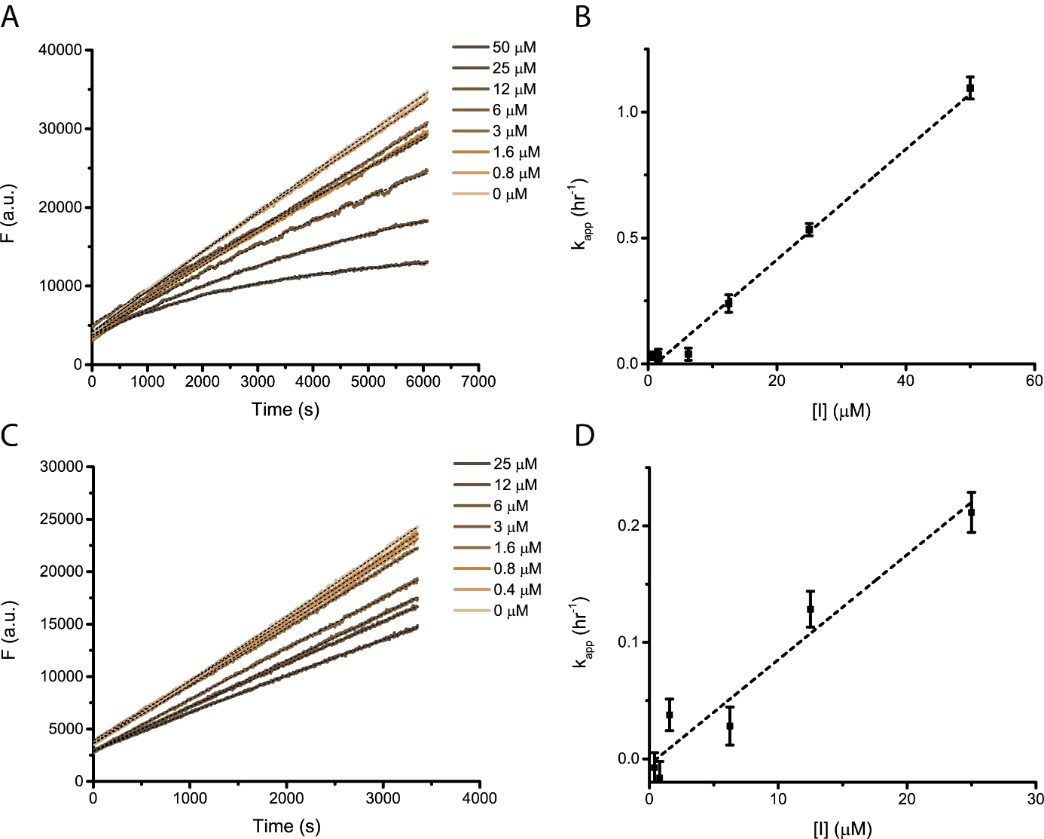


Figure S17: TlGH12A inhibition kinetics. A) Fluorescence over time for TlGH12A acting on 4MU-GG in the presence of variable concentrations of GGcyc. Exponential decay fit lines for each data set are shown as dashed black lines. B) Apparent decay constant (*k*_app_) vs. inhibitor concentration for TlGH12A inhibited by GGcyc. The linear fit used to calculate *k*_inact_/*K*_I_ is shown as a black dash line. Error bars represent the standard error in the *k*_app_ value determined from the fit of the line in A. C) Fluorescence over time for TlGH12A acting on 4MU-GG in the presence of variable concentrations of Biotin-ABP-Cel. Exponential decay fit lines for each data set are shown as dashed black lines. D) Apparent decay constant (*k*_app_) vs. inhibitor concentration for TlGH12A inhibited by Biotin-ABP-Cel. The linear fit used to calculate *k*_inact_/*K*_I_ is shown as a black dash line. Error bars represent the standard error in the *k*_app_ value determined from the fit of the line in C.

**Additional Tables**

Table S1: Basidiomycete strains used in this study.

| Strain | BRFM ID | Classification | Origin | Ref |
| --- | --- | --- | --- | --- |
| *Trametes gibbosa* | 952 | Basidiomycota, Polyporaceae | France, Ariège, Moulis, Beech Forest | (3) |
| *Polyporus brumalis* | 958 | Basidiomycota, Polyporaceae | France, Hautes Pyrénées, Puydarieux | (4) |
| *Abortiporus biennis* | 1215 | Basidiomycota, Meruliaceae | France, Orne | (5) |
| *Hexagonia nitida* | 1328 | Basidiomycota, Polyporaceae | France, Vaucluse | (5) |
| *Trametes ljublarskyi* | 957 | Basidiomycota, Polyporaceae | France | (5, 6) |
| *Leiotrametes menziesii* | 1557 | Basidiomycota, Polyporaceae | Martinique (Island) | (5) |
| *Fomes fomentarius* | 1323 | Basidiomycota, Polyporaceae | France, Corsica | (5) |
| *Trametes Meyenii* | 1361 | Basidiomycota, Polyporaceae | India | (5) |
| *Pycnoporus sanguineus* | 902 | Basidiomycota, Polyporaceae | Macouria, French Guyana. | (7) |
| *Leiotrametes sp.* | 1048 | Basidiomycota, Polyporaceae | French Guiana, Kourou | (3, 8) |

Table S2: Hydrolytic kinetics of LsGH5_5A, LsGH10A, and TlGH12A acting on 4MU-GG and 4MU-Xyl2

| Enzyme | Substrate | *K_M_* (μM) | *k_cat_* (s^-1^) | *k_cat_*/*K_M_* (s^-1^M^-1^) |
| --- | --- | --- | --- | --- |
| LsGH5_5A | 4MU-GG | ND | ND | 7.3±0.4 |
|  | 4MU-Xyl2 | ND | ND | <0.02 |
| LsGH5_7A | 4MU-GG | ND | ND | <0.02 |
|  | 4MU-Xyl2 | ND | ND | <0.02 |
| LsGH10A | 4MU-GG | ND | ND | 350±20 |
|  | 4MU-Xyl2 | 32+/-3 | 3+/-0.1 | 94000 |
| TlGH12A | 4MU-GG | 600+/-100 | 0.0015+/-0.0003 | 2.5 |
|  | 4MU-Xyl2 | ND | ND | <0.02 |

*ND = not determined

Table S3: Inhibition kinetics of LsGH5_5A, LsGH10A, and TlGH12A.

| Enzyme | Compound | *K*_I_ (μM) | | *k*_inact_ (hr^-1^) | | *k*_inact_/*K*_I_ (s^-1^M^-1^) |
| --- | --- | --- | --- | --- | --- | --- |
| LsGH5_5A | GGcyc | >100 | >2.6 | | 7.2±0.4 | |
|  | Biotin-ABP-Cel | >50 | >0.9 | | 4.9±0.8 | |
| LsGH10A | GGcyc | ND | ND | | 3±1 | |
|  | XXcyc | >6 | >560 | | 26000 | |
|  | Biotin-ABP-Xyn | 0.021+/-0.001 | <0.16 | | <2000 | |
| TlGH12A | GGcyc | >100 | >2.2 | | 6.2±0.5 | |
|  | Biotin-ABP-Cel | ND | ND | | 1.0±0.3 | |

*ND = not determined

Table S4: Sequences of enzymes produced recombinantly in *P. pastoris*.

| Enzyme name | Produced amino acid sequence |
| --- | --- |
| LsGH5_5A | QAPVWSQCGGTGWTGETTCATGSVCTALNPSYSQCVPGTGASSTPSATSSAPAPSASSTCAPNSPPSSAGKLRFAGVNISGFD  FGCGTDGTCTASGAWPPLTQYYGMDGAGQMKHFVEDDGFNVFRLPVGWQFLTNGAANGDIDEDNFTEYDALVQACLDSGASCI  VDVHNYARFNGKIIGQGGPTNDEFAALWSSLAAKYADNDKIIFGVMNEPHDVPDINLWAESVQAAVTAIRNAGATSQLILLPG  NNWTSAETFVSNGSADALNKVTNPDGSITGLIFDVHKYLDFDNSGTNAECTTNNIDNAWAPLAQWLRCNGRQAFNTETGGGNV  ASCEQFMCEQVAFQKANSDVFLGYVGWAAGNFYNGYVLSEVPTQNADGTWTDQPLVAQCLAPNANAVDHHHHHH |
| LsGH5_7A | AAPEWGQCGGIGWTGDTTCVAGTVCTVQNPYYSQCLPGVSSVPTSTPTTVPPTSTPSTPTSTSVPPPSSTGFVKVSGQKFVLN  GKTYPLVGANSYWVGLMGYSTAQMNQAFADIAATGATTVRTWGFNDVTTANGIYYQLWQNGKATVNTGATGLQNFDNVIAAAK  ANGLRLIVALTNNWSDYGGMDVYVQQIAGSPDHDLFYTNANVIAAYKSYIKTFVGRYVNEPTLLGWELANEPRCSGSTGTSTG  TCTTQTITKWASDISAYIKSIDSNHLVAIGDEGFFNEPGNPSYPYQGGEGIDFNANLNISTIDFGTAHLYPISWGQTSDPTGW  GSQWITDHATSQKAANKPVILEEFGVTDSQASTYTAWYNTVITSGLTGDLIWQAGSHFSTGSTPDDGYAIYPDDPVYPIESQH  FAAVKARGVDHHHHHH |
| LsGH10A | QSQEWGQCGGIGWTGAMTCVAGTVCTVLNPYYSQCLPGSASSTPTVPTSTPTTPTSTPGQPAPSGTGLNSLAKAAGKLYFGTA  TDNSELTDQAYTAILDNIKEFGQITPANSMKWDATEPTQGEFTFSGGDQIANLAKTNGQLLRGHNCVWYNQLPSWVSNGKFTA  AQLTDIIQTHCGTLVGHYKGQVYAWDVINEPFNDDGTWRSDVFYNTLGTDFVPIALQAARAADPNAKLYINDYNIEQTGAKAT  AMLNLVKQLKADGIPIDGVGFQCHFIVGEVPGSFQQVLEQFTALGVEVAITELDIRMTLPATQASLQQQQKDYQTVVQACMNV  EGCVGITVWDFTDKYSWVPSTFSGQGEACPWDQNLQKKLAYTGISTALSAVDHHHHHH |
| TlGH12A | QLITGQYDCLPAGAYTLCQNLWGKSSGVGAQNSTLLSASGNNVSWRTIWQWANNPNNVKSYANVEHNTAKGVQLSKLKSAPTA  WQWQYESQSNPIRADVSYDIWTGTTPTGNPASSASSFEIMIWLSGKGGIQPVGSQIQSGINLAGHNWNLWRGPNANWQVLSFV  SQDGDITNFNADLKEFFDYIVQNQGVSSSQFVQAIQTGTEPFTGSASLLTQNFNVALNQVDHHHHHH |

**Additional References**

1. de Boer C, McGregor NGS, Peterse E, Schröder SP, Florea BI, Jiang J, Reijngoud J, Ram AFJ, van Wezel GP, van der Marel GA, Codée JDC, Overkleeft HS, Davies GJ. 2020. Glycosylated cyclophellitol-derived activity-based probes and inhibitors for cellulases. RSC Chem Biol 1:148–155.

2. Ueno Y, Jose J, Loudet A, Pérez-Bolívar C, Anzenbacher P, Burgess K. 2011. Encapsulated energy-transfer cassettes with extremely well resolved fluorescent outputs. J Am Chem Soc 133:51–55.

3. Berrin JG, Navarro D, Couturier M, Olivé C, Grisel S, Haon M, Taussac S, Lechat C, Courtecuisse R, Favel A, Coutinho PM, Lesage-Meessen L. 2012. Exploring the natural fungal biodiversity of tropical and temperate forests toward improvement of biomass conversion. Appl Environ Microbiol 78:6483–6490.

4. Miyauchi S, Rancon A, Drula E, Hage H, Chaduli D, Favel A, Grisel S, Henrissat B, Herpoël-Gimbert I, Ruiz-Dueñas FJ, Chevret D, Hainaut M, Lin J, Wang M, Pangilinan J, Lipzen A, Lesage-Meessen L, Navarro D, Riley R, Grigoriev I V., Zhou S, Raouche S, Rosso M-N. 2018. Integrative visual omics of the white-rot fungus Polyporus brumalis exposes the biotechnological potential of its oxidative enzymes for delignifying raw plant biomass. Biotechnol Biofuels 11:201.

5. Zhou S, Raouche S, Grisel S, Navarro D, Sigoillot JC, Herpoël-Gimbert I. 2015. Solid-state fermentation in multi-well plates to assess pretreatment efficiency of rot fungi on lignocellulose biomass. Microb Biotechnol 8:940–949.

6. Paës G, Navarro D, Benoit Y, Blanquet S, Chabbert B, Chaussepied B, Coutinho PM, Durand S, Grigoriev I V., Haon M, Heux L, Launay C, Margeot A, Nishiyama Y, Raouche S, Rosso M-N, Bonnin E, Berrin J-G. 2019. Tracking of enzymatic biomass deconstruction by fungal secretomes highlights markers of lignocellulose recalcitrance. Biotechnol Biofuels 12:76.

7. Miyauchi S, Hage H, Drula E, Lesage-Meessen L, Berrin J-G, Navarro D, Favel A, Chaduli D, Grisel S, Haon M, Piumi F, Levasseur A, Lomascolo A, Ahrendt S, Barry K, LaButti KM, Chevret D, Daum C, Mariette J, Klopp C, Cullen D, de Vries RP, Gathman AC, Hainaut M, Henrissat B, Hildén KS, Kües U, Lilly W, Lipzen A, Mäkelä MR, Martinez AT, Morel-Rouhier M, Morin E, Pangilinan J, Ram AFJ, Wösten HAB, Ruiz-Dueñas FJ, Riley R, Record E, Grigoriev I V, Rosso M-N. 2020. Conserved white-rot enzymatic mechanism for wood decay in the Basidiomycota genus Pycnoporus. DNA Res 27:1–14.

8. Hage H, Miyauchi S, Virágh M, Drula E, Min B, Chaduli D, Navarro D, Favel A, Norest M, Lesage-Meessen L, Bálint B, Merényi Z, de Eugenio L, Morin E, Martínez AT, Baldrian P, Štursová M, Martínez MJ, Novotny C, Magnuson JK, Spatafora JW, Maurice S, Pangilinan J, Andreopoulos W, LaButti K, Hundley H, Na H, Kuo A, Barry K, Lipzen A, Henrissat B, Riley R, Ahrendt S, Nagy LG, Grigoriev I V., Martin F, Rosso MN. 2021. Gene family expansions and transcriptome signatures uncover fungal adaptations to wood decay. Environ Microbiol https://doi.org/10.1111/1462-2920.15423.
